# Caloric restriction protects from acute and chronic kidney injury by inhibiting monocyte recruitment

**DOI:** 10.1016/j.isci.2025.113094

**Published:** 2025-07-11

**Authors:** Paolo Molinari, Alberto Verlato, Johan Noble, Sara Alibrandi, Sofia Bin, Katja Ferrari, Paolo Malvezzi, Carlo Alfieri, Giuseppe Castellano, Laura Perin, Valter Longo, Paolo Cravedi

**Affiliations:** 1 Translational Transplant Research Center (TTRC), Icahn School of Medicine at Mount Sinai, New York, NY, USA; 2 Unit of Nephrology, Dialysis and Kidney Transplantation, Fondazione IRCCS Ca’ Granda Ospedale Maggiore Policlinico di Milano, Milan, Italy; 3 Nephrology, Hemodialysis, Apheresis and Kidney Transplantation Department, University Hospital Grenoble, 38043 Grenoble, France; 4 Univ. Grenoble Alpes, INSERM U, CNRS UMR, Institute for Advanced Biosciences, Grenoble, France; 5 Department of Medicine and Surgery, University of Parma, Parma, Italy; 6 Department of Clinical Sciences and Community Health, University of Milan, Milan, Italy; 7 GOFARR Laboratory for Organ Regenerative Research and Cell Therapeutics in Urology, Saban Research Institute, Division of Urology, Children’s Hospital Los Angeles (CHLA), Los Angeles, CA USA; 8 Longevity Institute, Leonard Davis School of Gerontology, University of Southern California, Los Angeles, CA 90089, USA; 9 Nephrology Unit, University Hospital of Parma, Parma, Italy; 10 Department of Medical and Surgical Sciences (DIMEC), Alma Mater Studiorum - University of Bologna, Bologna, Italy; 11 Nephrology, Dialysis and Kidney Transplant Unit, IRCCS Azienda Ospedaliero- University of Bologna, Bologna, Italy

**Keywords:** Immunology, Cell biology, Diet

## Abstract

Diet influences disease progression, yet the effects of fasting on acute kidney injury (AKI) and its transition to chronic kidney disease (CKD) remain unclear. This study evaluated fasting-mimicking diet (FMD) cycles versus *ad libitum* feeding in murine models of AKI and CKD induced by aristolochic acid or folic acid. FMD significantly reduced serum creatinine, kidney injury, and maladaptive repair marker expression, and promoted faster recovery. It also lowered renal cytokines and pro-fibrotic genes, reduced CCL2 levels, and decreased monocyte recruitment while favoring protective monocyte phenotypes. Cycles of caloric restriction yielded similar nephroprotection. Initiating FMD at the peak of AKI enhanced repair and attenuated inflammation. Inhibition of CCR2 abolished FMD’s protective effects, implicating the CCL2/CCR2 axis in mediating its benefits. However, broader anti-inflammatory actions may also contribute, and reduced CCL2 may reflect downstream effects. These findings highlight the potential of dietary interventions to modulate kidney injury and inflammation in AKI and CKD.

## Highlights

• Fasting-mimicking diet and caloric restriction reduce the severity of acute kidney injury
• When started at the peak of AKI, FMD accelerates kidney repair
• FMD reduces monocyte recruitment into the kidney, in part through CCL2/CCR2 axis
• FMD also lowers the renal expression of cytokines and pro-fibrotic markers

## Graphical abstract

**Figure:**
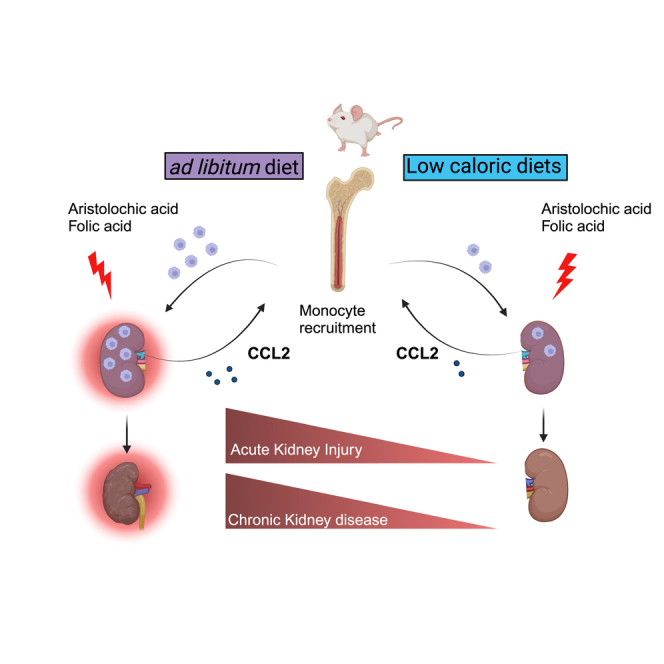

## Introduction

Acute kidney injury (AKI) occurs in up to 7% of hospitalized patients and in approximately one-third of patients in the intensive care unit (ICU).^1^ It increases the risk of mortality, and patients who survive an episode of AKI are more likely to progress to chronic kidney disease (CKD).^2^

The most frequent histological pattern of AKI is acute tubular necrosis (ATN), characterized by tubular epithelial cell death and dysfunction in one or several tubular segments.^3^^,^^4^ After acute renal injury, tubular cell death ensues from the activation of surrounding cells, leading to the production of damage-associated molecular pattern (DAMP) molecules and other alarmins.^4^^,^^5^^,^^6^^,^^7^ These signals recruit and activate innate immune cells, including circulating and resident macrophages.^4^^,^^8^^,^^9^ During the initial phases, activated macrophages exhibit robust phagocytic activity, allowing for the clearance of apoptotic bodies and necrotic cellular debris. In the later phases, macrophages secrete resolvins, lipoxins, transforming growth factor β (TGF-β), and matrix metalloproteinases, which specifically cleave chemokines and chemoattractants, allowing for the resolution of the inflammatory response and for kidney recovery. However, macrophages can also release proinflammatory cytokines, including tumor necrosis factor alpha (TNF-α), interleukin-1 (IL-1), and IL-6, which exacerbates the initial inflammatory response^10^^,^^11^^,^^12^ and promotes maladaptive repair of tubular cells, leading to renal scarring. Therefore, macrophages are critical modulators of AKI severity and transition to CKD.^13^^,^^14^^,^^15^^,^^16^^,^^17^^,^^18^

The number of macrophages in kidneys under normal conditions is limited. Upon renal injury, macrophages are recruited from the bone marrow and accumulate in the injured kidney.^10^^,^^11^^,^^13^^,^^14^^,^^17^^,^^18^^,^^19^ Monocyte chemoattractant protein-1 (MCP-1 or CCL2) is a critical chemokine involved in recruiting monocytes from the bone marrow to target organs, and pharmacological inhibition of CCL2 has been proposed as a strategy to reduce AKI severity.^20^^,^^21^^,^^22^^,^^23^

Hypocaloric diets or fasting regimens are associated with reduced inflammation and multisystem cell regeneration ^24^^,^^25^ and improved outcomes in metabolic, autoimmune, and inflammatory diseases,^18^^,^^24^^,^^26^^,^^27^ forming the rationale for testing their efficacy in models of AKI and transition to CKD. Notably, caloric restriction has been associated with reduced emigration of monocytes from the bone marrow both in mice and humans.^28^

Extended water-only fasting presents challenges for most individuals. FMD is a unique hypocaloric diet regimen characterized by cycles of low caloric diet lasting 5 days followed by a standard *ad libitum* diet.^29^ This diet regimen has been shown to be safer and better tolerated than other caloric restriction regimes and reduces the severity of a variety of inflammatory diseases both in mice and humans.^18^^,^^24^^,^^26^^,^^27^^,^^30^

Herein, we tested the hypothesis that FMD cycles reduce AKI severity and progression from AKI to CKD by inhibiting the recruitment of pathogenic monocytes into the kidney.

## Results

### FMD and low-caloric diet reduce acute and chronic AA-induced kidney function impairment in mice

Male BALB/c mice received FMD, low caloric (Low Cal), or *ad lib* diet, starting one week before aristolochic acid (AA) administration (Figures S1A and S1B). In mice without AA injection, FMD cycles and Low Cal regimen were not associated with renal histological changes (Figure S2A). BUN and serum creatinine decreased during the first 5 days of low caloric intake, but then these parameters return to baseline at the end of the 2 days of refeeding (Figure S2B). Therefore, we always measured blood urea nitrogen (BUN) and serum creatinine at the end of each FMD cycle. During FMD and Low Cal cycles, mice progressively lose weight but returned to baseline during the refeeding phase (Figure S2C).

**Figure 1:**
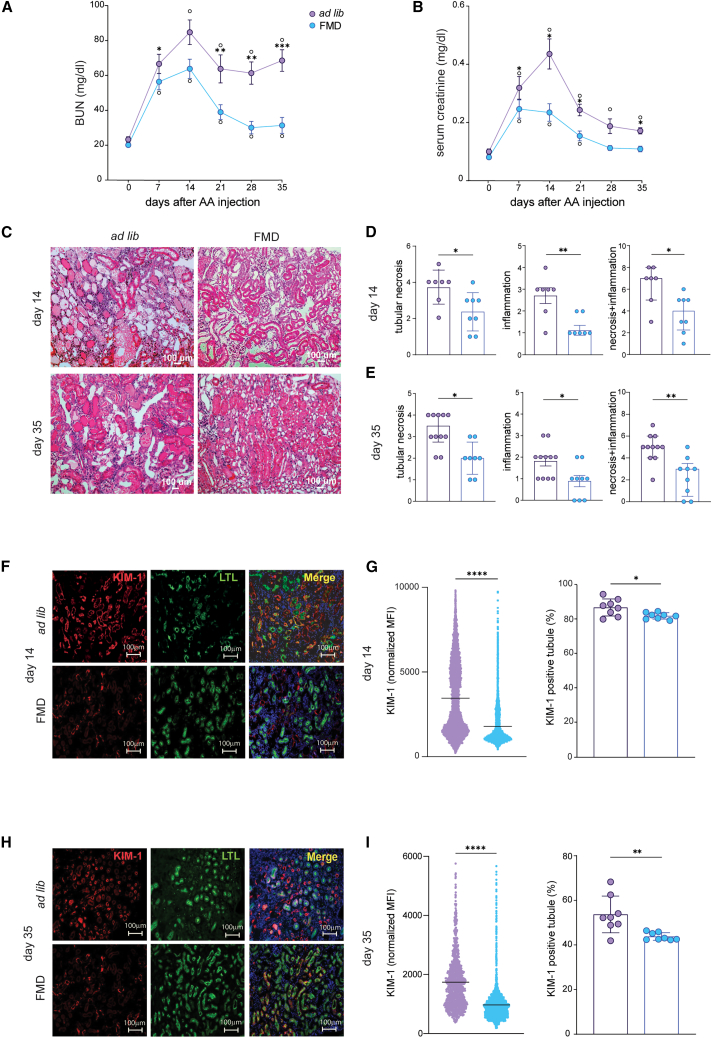
FMD reduces renal damage 14 and 35 days after AA injection (A and B) (A) Blood urea nitrogen (BUN) and (B) serum creatinine values at serial time points in BALB/c male mice injected with AA on *ad lib* diet (*n* = 13) or FMD (*n* = 18). (C) Representative brightfield images (H&E staining) of *ad lib* and FMD mice cortical tubular sections at 14 and 35 days after AA injection. A scale bar representing 100 μm is shown in the lower right corner. (D and E) Bar plots of histologic scores of tubular injury and inflammation severity at 14 days (*ad lib*, *n* = 7; FMD, *n* = 8) and at 35 days (*ad lib*, *n* = 11; FMD, *n* = 9) after AA injection. (F and G) Representative immunofluorescence (IF) images of KIM-1 (red), LTL (green), and DAPI (blue) cortical tubular staining and data quantification in *ad lib* and FMD mice at 14 days after AA injection (*ad lib*, *n* = 8; FMD, *n* = 8). A scale bar representing 100 μm is shown in the lower right corner. (H and I) Representative IF images of KIM-1 (red), LTL (green), and DAPI (blue) cortical tubular staining and data quantification in *ad lib* and FMD mice at 35 days after AA injection (*ad lib*, *n* = 8; FMD, *n* = 8). A scale bar representing 100 μm is shown in the lower right corner. Repeated measures ANOVA model was used to assess statistical significance at different time points (A and B). t test was used to compare distributions between groups at the same time point. Data are represented as mean ± SEM ○: *p* < 0.05 vs. baseline; ∗*p* < 0.05, ∗∗*p* < 0.01, ∗∗∗*p* < 0.001, and ∗∗∗∗*p* < 0.0001 vs. *ad lib* at the same time point; ns: not significant.

Mice in the *ad lib* group developed AKI, peaking at 2 weeks post-AA injection and partially recovered kidney function by day 35, while mice on FMD had significantly less acute injury and almost fully recovered from kidney damage, as shown by trends in BUN and serum creatinine levels (Figures 1A and 1B). Mice on FMD had significantly lower BUN and serum creatinine levels from day 14–35 compared to *ad lib* diet mice (Figures 1A and 1B). Differences in renal function were paralleled by less severe tubular necrosis and inflammation, both at 14 and 35 days in FMD mice and Low Cal mice (Figures 1C–1E, S3A, and S3B). FMD mice also displayed significantly lower expression of kidney injury molecule-1 (KIM-1), a tubular injury marker,^31^ than *ad lib* controls both at 14 and 35 days (Figures 1F–1I).

Following AA administration, mice on *ad lib* diet experienced progressive weight loss until day 14, followed by gradual recovery to baseline values by day 35 (Figure S2D). At the end of each FMD cycle, mice reached approximately 15% reduction of their initial weight, promptly recovering during the final two days of the cycle when they had access to the regular diet (Figure S2D). By day 35, the weight of FMD mice averaged 10% higher than baseline, akin to the natural age-related weight increase in mice of that age (Figure S2D).^32^ Conversely, mice on *ad lib* diet failed to recover weight to the extent observed in physiologically aging mice (Figure S2D).

These data indicate that FMD and Low Cal diets are well tolerated and reduce severity of AA-induced AKI and progression to CKD.

### FMD reduces renal expression of proinflammatory and pro-fibrotic genes

Kidney damage is mediated by immune infiltrates and injured tubular cells releasing cytokines and pro-inflammatory factors, which exacerbate tubular cell damage and inhibit tissue repair. FMD significantly reduced the expression of key proinflammatory markers, including IL*-1β*, *IL-6*, and *TNF-α*^33^^,^^34^ at 14 and 35 days post-AA injection (Figures S4A–S4C). FMD also reduced expression of *CTGF*, *TGFβ*, and *IL-33* genes, that promote extracellular matrix deposition, fibrosis,^35^ and maladaptive repair^36^ (Figures S4D–S4F). In contrast, renal expression of epidermal growth factor (EGF), crucial for tubular repair^37^ was significantly increased in FMD mice 35 days after AA injection (Figure S4G). Therefore, FMD lowers the expression of pro-inflammatory and pro-fibrotic cytokines and markers of maladaptive repair while enhancing reparative stimuli at the renal level.

### FMD reduces intrarenal immune infiltrates and migratory macrophages

**Figure 2:**
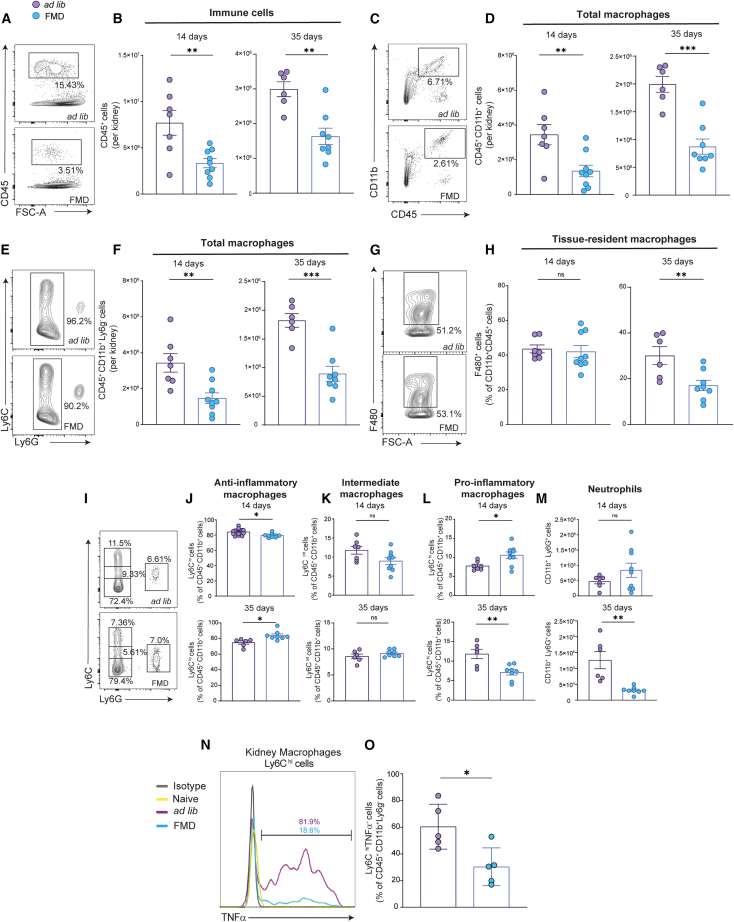
FMD reduces kidney infiltrates and pro-inflammatory cytokines (A and B) representative contour plot at 14 days and kidney quantification (bar plot) of CD45^+^ cells immune cells at 14 and 35 days in *ad lib* (*n* = 7) and FMD mice (*n* = 9). (C and D) Representative contour plot of CD45^+^CD11b^+^ myeloid cells in *ad* (*n* = 7) and FMD mice (*n* = 9). (E and F) Representative contour plot of CD45^+^CD11b^+^Ly6G^−^ macrophages cells at 14 and 35 days in *ad lib* (*n* = 7) and FMD mice (*n* = 9). (G and H) Representative contour plot and kidney quantification (bar plot) of CD45^+^ CD11b^+^F480^+^ macrophage cells at 14 and 35 days in *ad lib* (*n* = 7) and FMD mice (*n* = 9). (I–M) Representative contour plot and kidney quantification (bar plot) of CD45^+^CD11b^+^Ly6G^−^Ly6C^hi,int,low^ macrophages and CD45^+^CD11b^+^Ly6G^+^ neutrophils at 14 and 35 days in *ad lib* and FMD mice (*ad lib*, *n* = 7 at 14 days and *n* = 6 at 35 days; FMD, *n* = 9 at 14 days and *n* = 8 at 35 days). (N and O) Representative histogram and data quantification of TNFα positive pro-inflammatory macrophages (Ly6C^hi^) in the spleen in *ad lib* and FMD mice (*ad lib*, *n* = 5 and for FMD, *n* = 5) at 35 days. t test was used to compare distributions between groups at the same time point. Data are represented as mean ± SEM ∗*p* < 0.05, ∗∗*p* < 0.01, ∗∗∗*p* < 0.001, and ∗∗∗∗*p* < 0.0001 vs. *ad lib* at the same time point; ns: not significant.

FMD significantly reduced total intrarenal immune cells (CD45^+^) compared to *ad lib* diet, both at 14 days and at 35 days after AA injection (Figures 2A and 2B). Total intrarenal macrophages (CD45^+^CD11b^+^Ly6G^−^) were significantly lower (both in absolute numbers and as a percentage of infiltrating immune cells) during AKI and during the transition to CKD in FMD mice (Figures 2C–2F and S5A). In the absence of AA-induced injury, the percentage of intrarenal macrophages was similar between FMD and Ad lib diets at 35 days (Figure S5B). FMD did not affect the percentage of F4/80^+^ tissue-resident renal macrophages at 14 days, but it significantly reduced them at 35 days (Figures 2G and 2H).

In mice, two subpopulations of monocytes exist: Ly6C^hi^ monocytes that act in the first phase of damage and exhibit robust phagocytic activity,^13^^,^^38^ allowing for the clearance of apoptotic bodies and necrotic cellular debris, and Ly6C^low^ monocytes, involved in late responses to inflammation and tissue repair.^39^ As a result of the overall reduced number of macrophages in the kidneys of mice on FMD at both 14 and 35 days after AA, both proinflammatory Ly6C^hi^ and anti-inflammatory Ly6C^low^ macrophages declined in the kidneys of FMD mice. Within the CD11b^+^ macrophage compartment, the percentages of Ly6C^hi^ macrophages were initially higher, and then progressively declined at 35 days in FMD mice, while Ly6C^low^ macrophages followed an opposite trend (Figures 2I–2L). At 35 days, total intrarenal neutrophils (CD45^+^CD11b^+^Ly6G^+^) were significantly lower in FMD-treated mice compared to *ad lib* (Figure 2M). Of note, LyC6^hi^ macrophages isolated from the kidneys of FMD mice at 14 days after AA treatment showed significantly lower expression of TNFα (Figures 2N–2O).

FMD had no effects on the major immune cell subsets in the spleen at 14 and 35 days post-AA injection (Figures S5C–S5L).

Overall, these findings document that, during AKI and AKI to CKD transition, FMD reduces the recruitment of immune cells, particularly monocytes, into the kidney and promotes their anti-inflammatory/repair features.

### FMD reduces levels of CCL2 and circulating monocytes

**Figure 3:**
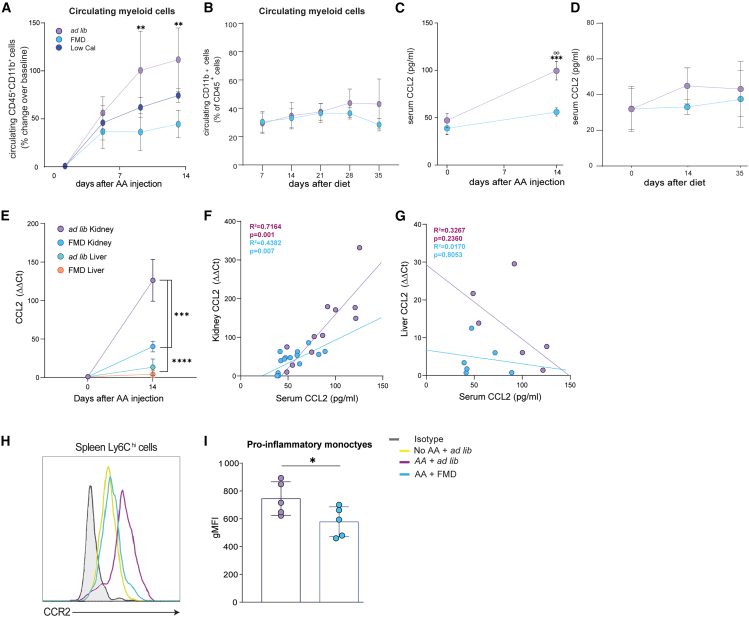
FMD reduces CCL2 and monocytes recruitment at 14 days after AA injection (A) Circulating CD45^+^CD11b^+^ myeloid cells changes (%) over baseline at 7 and 14 (*ad lib n* = 10, FMD *n* = 7, Low Cal *n* = 8) days after AA injection. (B) Circulating CD45^+^CD11b^+^ myeloid cells changes (%) over baseline at 7, 14, 21, 28, and 34 (*ad lib n* = 4, FMD *n* = 4) days after diet (FMD versus *Ad lib*) without AA. (C) Serum CCL2 levels (pg/mL) at 0 and 14 days (*ad lib n* = 18, FMD *n* = 12) after AA injection. (D) Serum CCL2 levels (pg/mL) at 0, 14 and 35 days after diet (FMD versus *Ad lib*) without AA (*ad lib n* = 4, FMD *n* = 4). (E) *CCL2* mRNA levels in kidney and liver in *ad* lib and FMD mice 14 days after AA injection (*ad lib n* = 6, FMD *n* = 8, *ad lib* liver *n* = 6, FMD liver *n* = 8). (F) Linear regression analysis of serum CCL2 levels and kidney *CCL2* transcripts 14 days after AA injection. (G) Linear regression analyses between serum CCL2 levels and liver *CCL2* transcripts 14 days after AA injection. (H and I) (H) Representative histogram and (I) quantification of CCR2 expression in spleen CD45^+^CD11b^+^Ly6G^−^Ly6C^hi^ pro-inflammatory monocytes; fluorescence was evaluated as gMFI (geometric mean fluorescent index) in *ad lib* vs. FMD mice after AA injection (*ad* lib *n* = 5, FMD *n* = 5). Repeated measures ANOVA model was used to assess statistical significance at different time points (A, B, C, and D). Correlation and linear regression analyses were performed to measure association. t test was used to compare gMFI distributions between groups at the same time point (Panel I). Data are represented as mean ± SEM ○: *p* < 0.05 vs. baseline; ○○ *p* < 0.01 vs. baseline ∗*p* < 0.05, ∗∗*p* < 0.01, ∗∗∗*p* < 0.001, and ∗∗∗∗*p* < 0.0001 vs. *ad lib* at the same time point; ns, not significant.

FMD and Low Cal diet significantly reduced circulating CD45^+^CD11b^+^ monocytes after AA-induced AKI (Figure 3A). In the absence of AA-induced injury, the low-caloric diet had no effect on circulating monocytes up to 35 days (Figure 3B). CCL2 is a crucial mediator of monocyte recruitment from the bone marrow.^28^^,^^40^ Therefore, we tested the hypothesis that FMD reduces AKI severity by reducing monocyte infiltration in the kidney through a CCL2/CCL2 receptor (CCR2R)-mediated mechanism.

FMD prevented increase in serum CCL2 levels at 14 days after AA injection (Figure 3C) while it had no effect on serum CCL2 in in mice that did not receive AA (Figure 3D). During AKI, *CCL2* gene expression sharply increased in the kidney, but not in the liver, an increase that was markedly reduced in FMD compared to *ad lib* mice (Figure 3E). We observed a significant correlation between serum CCL2 levels and renal, but not liver *Ccl2* mRNA expression (Figures 3F and 3G) both in FMD and *ad lib* mice. Of note, AA increased CCR2 expression in circulating monocytes, a phenomenon that was almost entirely prevented by FMD (Figures 3H and 3I).

Overall, this supports the notion that the kidney is the main source of CCL2 after injury and that increased CCL2 production leads to increased monocyte recruitment to the kidney, a mechanism that is counteracted by FMD.^28^

### Uncoupling CCL2/CCR2 signaling mimics FMD protective effects

To test for a causal link between FMD-induced CCL2 decline, reduced renal monocyte recruitment, and kidney failure, we treated FMD or *ad lib* BALB/c male mice with a CCR2 inhibitor (CCR2i) after AA injection (Figure S1B).

**Figure 4:**
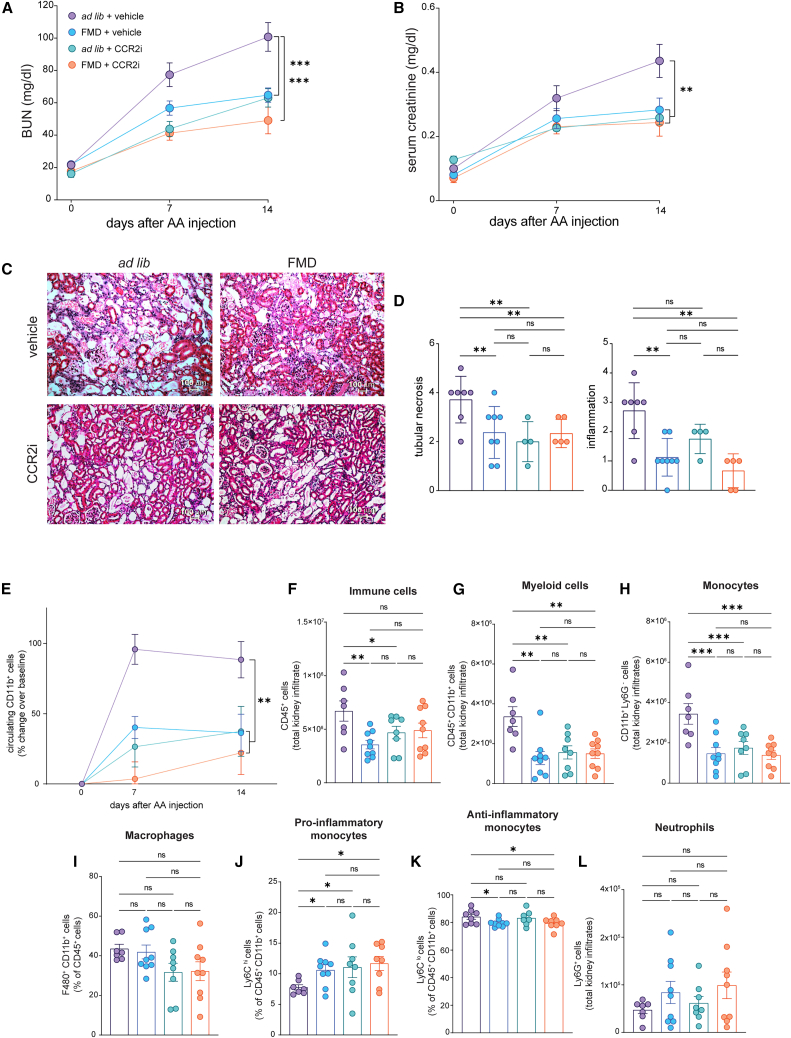
Renal damage, circulating monocytes, and renal infiltrating monocytes at 14 days after AA injection with or without CCR2i (A and B) (A) Blood urea nitrogen (BUN) and (B) serum creatinine values at serial time points after AA injection (*ad lib* + vehicle *n* = 10, FMD + vehicle *n* = 7, *ad lib* + CCR2i *n* = 8, FMD + CCR2i *n* = 9); mice were injected with CCR2i or vehicle every day starting from the day of AA injection till day 14. (C) Representative brightfield images (H&E) of *ad lib* + vehicle, FMD + vehicle, *ad lib* + CCR2i and FMD + CCR2i of kidney cortical tubular sections at day 14 after AA injection. A scale bar representing 100 μm is shown in the lower right corner. (D) Bar plot of histologic scores of tubular injury and inflammation severity at 14 days (*ad lib* + vehicle *n* = 7, FMD + vehicle *n* = 8, *ad lib* + CCR2i *n* = 4, FMD + CCR2i *n* = 5). (E) Circulating CD45^+^ CD11b^+^ myeloid cell changes (%) over baseline at 7 and 14 days (*ad lib n* = 10, FMD *n* = 7, *ad lib* + CCR2i *n* = 8, FMD + CCR2i *n* = 9). (F) Kidney quantification of CD45^+^ immune cells at 14 days (*ad lib n* = 7, FMD *n* = 9, *ad lib* + CCR2i *n* = 8, FMD + CCR2i *n* = 9). (G) Kidney quantification of CD45^+^ CD11b^+^ myeloid cells at 14 days (*ad lib n* = 7, FMD *n* = 9, *ad lib* + CCR2i *n* = 8, FMD + CCR2i *n* = 9). (H) Kidney quantification of CD45^+^CD11b^+^Ly6G^−^ monocyte cells at 14 days (*ad lib n* = 7, FMD *n* = 9, *ad lib* + CCR2i *n* = 8, FMD + CCR2i *n* = 9). (I) Kidney quantification of CD45^+^ CD11b^+^F480^+^ macrophage cells at 14 days (*ad lib n* = 7, FMD *n* = 9, *ad lib* + CCR2i *n* = 8, FMD + CCR2i *n* = 9). (J) Kidney quantification of CD45^+^CD11b^+^Ly6G^−^Ly6C^high^ pro-inflammatory monocyte cells at 14 days (*ad lib n* = 7, FMD *n* = 9, *ad lib* + CCR2i *n* = 8, FMD + CCR2i *n* = 9). (K) Kidney quantification of CD45^+^CD11b^+^Ly6G^−^Ly6C^low^ anti-inflammatory monocyte cells at 14 days (*ad lib n* = 7, FMD *n* = 9, *ad lib* + CCR2i *n* = 8, FMD + CCR2i *n* = 9). (L) Kidney quantification of CD45^+^CD11b^+^Ly6G^+^ neutrophil cells at 14 days (*ad lib n* = 7, FMD *n* = 9, *ad lib* + CCR2i *n* = 8, FMD + CCR2i *n* = 9). Repeated measures ANOVA model was used to assess statistical significance at different time points. One-way ANOVA was used to compare the 4 groups at the same time point. Data are represented as mean ± SEM ∗*p* < 0.05, ∗∗*p* < 0.01, ∗∗∗*p* < 0.001, and ∗∗∗∗*p* < 0.0001 vs. *ad lib* at the same time point; ns not significant.

A*d lib* mice treated with CCR2i had significantly lower BUN and serum creatinine at 14 days after AA injection compared to *ad lib* mice treated with vehicle (Figures 4A and 4B). In the presence of CCR2i, FMD had no additional protective effect on kidney function (Figures 4A and 4B) or histological lesions (tubular necrosis, inflammation, and macrophages infiltration) after AA induced AKI (Figures 4C, 4D, and S6). Furthermore, no differences were found between the *ad lib* + CCR2i and FMD + CCR2i groups in renal function (Figures 4A and 4B) or histological severity indices (Figures 4C and 4D). This suggests that FMD exerts its renoprotective effects mainly by inhibiting CCL2/CCR2 axis. The immunological changes described in FMD mice (Figure 2) were recapitulated by CCR2i treatment and, in CCR2i treatment animals, FMD had no further effects (Figures 4E–4L).

These results support the idea that modulation of the CCL2/CCR2 axis contributes to the renoprotective effects of FMD. However, it remains possible that FMD and caloric restriction exert broader anti-inflammatory effects that result in downstream reductions in chemokines, including CCL2, and their receptors. In this context, the CCL2/CCR2 axis may reflect one of several parallel or downstream pathways rather than being the primary mediator. Additional studies will be required to determine whether the suppression of this chemokine axis is a direct, central mechanism of FMD action or a consequence of broader immunometabolic reprogramming.

### FMD inhibits mTOR and proliferation of kidney immune infiltrates

Caloric restriction increases AMPK activity, with anti-inflammatory and anti-proliferative effects.^26^ Mechanistic target of rapamycin (mTOR), a key regulator of immune cell function, is inhibited by AMPK,^41^ therefore, we tested mTOR activity in immune cells in the kidney after FMD. Levels of pAKT (upstream kinase of mTOR)^42^ were significantly reduced in renal infiltrating CD45^+^CD11b^+^ myeloid cells in FMD mice (Figures S7A and S7B). mTOR is known to promote cell proliferation.^41^ Accordingly, the expression of Ki67 proliferation marker significantly decreased after 5 days of FMD in renal immune cells overall (Figures S7C and S7D), including myeloid cells (Figures S7E and S7F).

Altogether, these results suggest that FMD does not only inhibit monocyte recruitment from the bone marrow, but it also inhibits their proliferation inside the kidney.

### FMD facilitates renal recovery from AKI

When started before AA administration, FMD reduced both AKI and CKD severity (Figure 1). However, the beneficial effects of FMD on CKD progression may be due to the reduced AKI.

**Figure 5:**
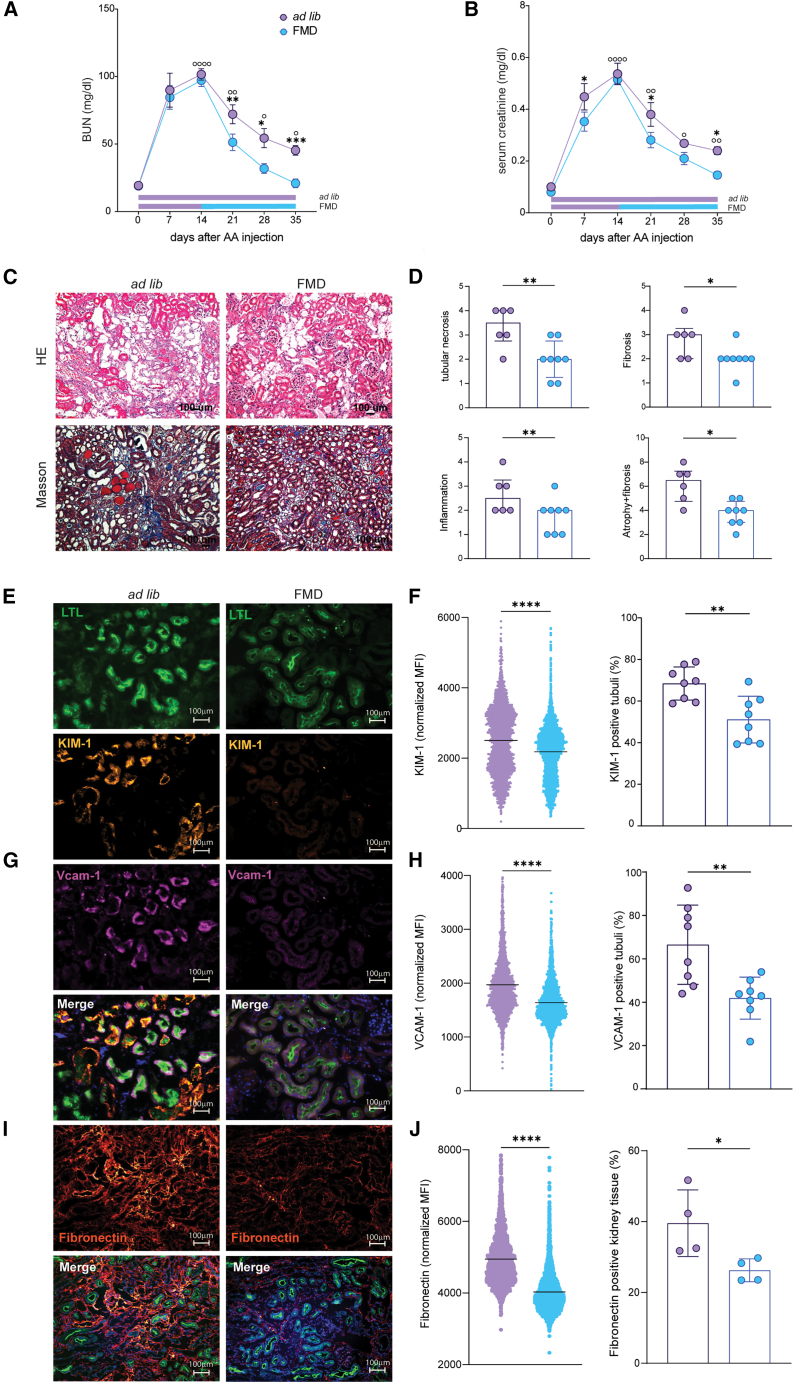
FMD-induced renal recovery after AKI in *ad lib* and FMD mice (A and B) (A) Blood urea nitrogen (BUN) and (B) serum creatinine values at serial time points after AA injection in *ad lib* (*n* = 6) and FMD (*n* = 8) BALB/c male mice until day 35. (C) Representative brightfield images of *ad lib* and FMD mice cortical tubular sections at different time points (upper quadrants H&E staining, lower quadrants Masson trichrome staining). A scale bar representing 100 μm is shown in the lower right corner. (D) Bar plot of histologic scores of tubular injury, inflammation, and fibrosis severity at 35 days (*ad lib*, *n* = 6 and FMD, *n* = 8). (E, G, and I) Representative IF images of KIM-1 (orange), VCAM-1 (magenta), fibronectin (red) LTL (green) and DAPI (blue) cortical tubular staining in *ad lib* and FMD mice at 35 days. A scale bar representing 100 μm is shown in the lower right corner. (F, H, and J) KIM-1, VCAM-1, and fibronectin MFI quantification in cortical tubules (left) and % of cortical tubuli positive (right) (positivity assessed by comparing tubular KIM-1 MFI with unstained control) at 35 days (*ad lib*, *n* = 8 and FMD, *n* = 8). Repeated measures ANOVA model was used to assess statistical significance at different time points (A and B). t test was used to compare distributions between groups at the same time point. Data are represented as mean ± SEM ○: *p* < 0.05 vs. baseline, ○○: *p* < 0.01 vs. baseline; ∗*p* < 0.05, ∗∗*p* < 0.01, ∗∗∗*p* < 0.001, and ∗∗∗∗*p* < 0.0001; ns, not significant.

To formally test the effects of FMD on renal recovery after AKI, we started FMD two weeks after AA administration (peak of renal injury) in BALB/c male mice (Figure S1C). Despite comparable AKI severity, FMD-treated mice had significantly lower BUN and serum creatinine levels during CKD transition (day 21–35) compared to *ad lib* mice (Figures 5A and 5B). At 35 days after AA injection, kidney function parameters returned to baseline in FMD mice, while they remained significantly higher in *ad* lib mice (Figures 5A and 5B).

At 35 days after AA injection, FMD mice displayed lower scores for tubular atrophy, inflammation, and fibrosis (Figures 5C, 5D, S8A, and S8B) and reduced expression of KIM-1 (Figures 5E and 5F), VCAM1 (a surface integrin expressed by tubular cells during maladaptive repair)^43^ (Figures 5G and 5H), and fibronectin (a key molecule for extracellular matrix deposition and subsequent fibrosis development) (Figures 5I and 5J).^44^

When initiated at the peak of AKI severity, FMD significantly reduced the recruitment of total immune cells (CD45^+^) to the kidney (Figures S9A and S9B), including the absolute numbers and percentages of myeloid cells (CD45^+^CD11b^+^) (Figures S9C and S9D) and monocytes (CD45^+^CD11b^+^Ly6G^−^) (Figures S9E and S9F) infiltrating the kidney 35 days after the onset of pathology. No significant differences were observed between *ad lib* and FMD mice in other myeloid cell populations infiltrating the kidney (Figures S9G–S9J).

FMD also significantly reduced gene expression of major pro-inflammatory cytokines (*IL1β*, *IL6*, and *Tnfα*)^33^^,^^34^ (Figures S9K–S9M), mediators of fibrosis (TGF-β, CTGF) (Figures S9N and S9O),^35^ and maladaptive repair (IL-33) (Figure S9P).^36^

These results provide evidence of FMD’s ability to mitigate renal injury and modulate immune cell populations even when initiated after the full development of injury.

### FMD reduces acute AA and folic acid induced kidney injury in B6 and BALB/c mice

**Figure 6:**
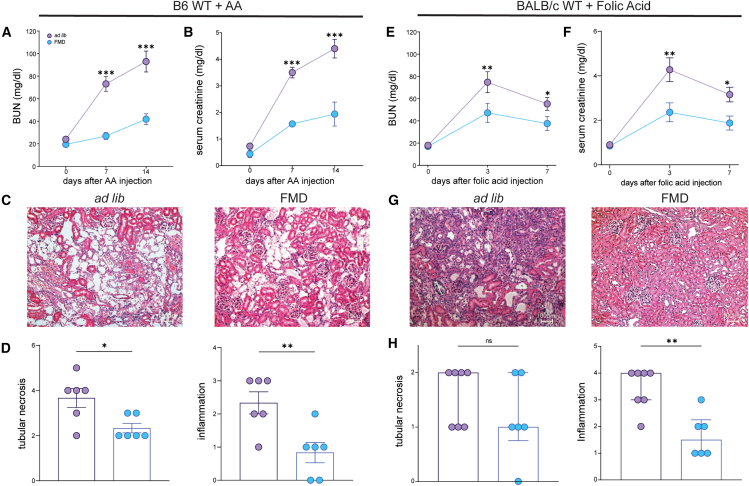
*Ad lib* and FMD diet in B6 mice receiving AA and in BALB/c mice treated with folic acid (A and B) (A) Blood urea nitrogen (BUN) and (B) serum creatinine values at day 7 and day 14 after AA injection in *ad lib* (*n* = 6) and FMD (*n* = 6) B6 male mice. (C) Representative brightfield images of *ad lib* and FMD mice cortical tubular sections (H&E staining). A scale bar representing 100 μm is shown in the lower right corner. (D) Bar plot of histologic scores of tubular injury and inflammation at 14 days in *ad lib* (*n* = 6) and FMD (*n* = 6). (E and F) (E) Blood urea nitrogen (BUN) and (F) serum creatinine values at day 3 and day 7 after folic acid injection in *ad lib* (*n* = 7) and FMD (*n* = 6) BALB/c male mice. (G) Representative brightfield images of *ad lib* and FMD mice cortical tubular sections (H&E staining). A scale bar representing 100 μm is shown in the lower right corner. (H) Bar plot of histologic scores of tubular injury and inflammation at 7 days in *ad lib* (*n* = 6) and FMD (*n* = 6). Repeated measures ANOVA model was used to assess statistical significance at different time points (A, B, E, and F). t test was used to compare distributions between groups at the same time point. Data are represented as mean ± SEM ○: *p* < 0.05 vs. baseline, ○○: *p* < 0.01 vs. baseline; ∗*p* < 0.05, ∗∗*p* < 0.01, ∗∗∗*p* < 0.001, and ∗∗∗∗*p* < 0.0001; ns, not significant.

We next tested whether FMD had protective effects on AA-induced AKI in male B6 mice, which are known to be more resistant to toxic damage than BALB/c (Figure S1B).^45^ FMD significantly reduced BUN and serum creatinine at 7 days after AA injection, peak of the acute kidney injury (Figures 6A and 6B). Kidneys from FMD mice also showed marked reduction of acute tubular damage and inflammatory infiltrates compared to *ad lib* controls (Figures 6C and 6D).

Finally, we tested the efficacy of FMD in another model of toxic AKI, induced by folic acid (Figure S1D). Folic acid, unlike AA which directly targets tubular cells, induces acute interstitial nephritis and subsequent AKI.^46^ Also in this model, FMD markedly reduced the severity of AKI, as indicated by lower BUN and serum creatinine values at 14 days (Figures 6E and 6F). FMD was also associated with a significant reduction in the severity of interstitial nephritis at the histological level (Figures 6G and 6H).

These data confirm that the beneficial effects of FMD on renal function and histology are reproducible in mice of different genetic backgrounds and in various disease models.

## Discussion

Our data show that FMD mitigates the severity of toxin-induced AKI in mice and protects against the development of chronic kidney disease. These findings are in line with prior evidence suggesting nephroprotective effects of caloric restriction.^47^^,^^48^ In particular, studies by others showed that caloric restriction reduces oxidative stress and reticulum stress while it protects mitochondria of tubular epithelial cells^49^^,^^50^^,^^51^ and favor tubular regeneration^37^ during both the acute and chronic phases. Of note, our study followed rigorous standards, including blinding of sample and data analyzers to diet allocation, the use of large sample sizes, and unbiased data quantification, which increases the reproducibility and reliability of our findings.^52^

Under physiological conditions, the number of macrophages in the kidney is limited, and those accumulating in the kidney upon injury are primarily derived from the bone marrow.^8^^,^^13^^,^^53^^,^^54^ Monocyte chemoattractant protein-1 (MCP-1 or CCL2) is a critical chemokine involved in recruiting monocytes from the bone marrow to target organs.^20^^,^^22^^,^^38^ Herein, we found that, upon AA injury, the kidney significantly upregulates *CCL2* gene expression, leading to a marked increase in serum CCL2 levels, which is almost entirely prevented by FMD. Notably, the liver contributed only minimally to CCL2 serum levels. This supports previous evidence that CCL2 is produced by various organs following injury and highlights that FMD can reduce it selectively in injured organs.^28^^,^^55^ From a molecular standpoint, prior data showed that fasting activates AMPK and PPARα, leading to reduced CCL2 production.^28^ Of note, we also newly found that FMD reduced CCR2 expression on monocytes, which likely contributes to the reduced monocyte emigration from bone marrow after kidney injury. Consistently, we found that the anti-inflammatory effects of FMD were more evident on infiltrating, rather than tissue resident, F4/80+ macrophages. Selective inhibition of CCR2 recapitulated all the renal and immunological effects of FMD and, in mice on CCR2 inhibition, FMD had no additional effects. Although this finding does not definitively prove that CCR2/CCL2 signaling mediates the protective effects of FMD or caloric restriction, it strongly suggests that most of the effects of FMD are mediated by inhibition of CCL2/CCR2 axis.

Together with inhibiting monocyte recruitment into the kidney, FMD also reduced macrophage activation, including TNF-α production in Ly6C^hi^ monocytes. FMD also favored the polarization of monocytes toward increased Ly6C^hi^ in the acute phase and Ly6C^lo^ in the chronic phase, a balance that has been shown by others to facilitate damage resolution.^39^

Fasting may also reduce monocyte numbers in the kidney by inducing their return from the bloodstream to the bone marrow. This process is orchestrated by the hypothalamic-pituitary-adrenal (HPA) axis-dependent release of corticosterone, which increases the expression of the CXCR4 chemokine receptor, facilitating monocyte retention in the bone marrow during nutrient scarcity. By reducing the number of monocytes available to infiltrate tissues, fasting can decrease inflammation and promote tissue repair.^56^

Of note, circulating monocytes and CCL2 levels in the serum did not significantly change when mice were exposed to multiple cycles of FMD in the absence of renal injury. This suggests that FMD immune modulating effects are less evident when there is no organ damage.

The efficacy of FMD was validated in both BALB/c and B6 strains. We also validated our results in an independent model of folic acid AKI in BALB/c male mice.^46^ In this model, FMD has confirmed its beneficial role in reducing the development of AKI and the progression of damage, both biochemically (BUN, sCr) and histologically. This was also associated with a reduction in monocytic infiltration at the renal level. In ischemia-reperfusion injury model, preoperative fasting was also beneficial to reduce AKI, improved mice survival,^57^^,^^58^ reduce inflammation, macrophages infiltration, and tubular apoptosis as compared to unlimited diet.^59^

Our studies did not test the impact of FMD on gut microbiome. Prior evidence showed that FMD modulates microbiota,^60^ which, on turn, could affect acute^61^ and chronic^62^ kidney injury. Further research is needed to test the impact of microbiome in mediating nephroprotective effects of FMD.

FMD was well tolerated. Sudden weight loss induced by FMD was rapidly recovered after only 2 days of refeeding. Importantly, the final weight of FMD mice at 35 days post-AA injection was significantly higher than that of *ad lib* mice, indicating the overall wellness of the mice.

Recently, the effects of caloric restriction have been tested in multiple human conditions, including, among others, type 2 diabetes, cardiovascular diseases, and cancer.^63^ Limited data exist on the effects of fasting regimens on acute and chronic kidney disease. Human trials have highlighted the benefits of caloric restriction on multiple outcomes, including AKI development in critically ill patients.^64^^,^^65^^,^^66^ Still, the evidence on the beneficial effects of calorie restriction regimens on AKI development and on the progression from AKI to CKD is limited. Our present data suggest that caloric restriction regimens may have a beneficial effect on the chronic progression of AKI.^67^

While fasting has notable health benefits, it can be challenging for many to adhere to due to its restrictive nature. The FMD offers a more feasible alternative, providing similar benefits while allowing for some nutrient intake. FMD has been tested in humans without showing significant side effects. The most commonly used protocol in these trials involved subjecting patients to one cycle of FMD per month, followed by a normal diet, for three consecutive months. This approach ensured that the caloric restriction regimen was well-tolerated and non-disruptive for the patients, while preserving its beneficial effects, in some preliminary trials.^18^^,^^24^^,^^25^^,^^26^^,^^27^^,^^30^ Most importantly, FMD has demonstrated beneficial and protective effects similar to those reported for caloric restriction in various settings, both in healthy individuals and in patients affected by inflammatory conditions. Particularly noteworthy are its effects in oncology, where it has shown the most striking benefits so far.^18^^,^^24^^,^^25^^,^^26^^,^^27^^,^^30^^,^^68^^,^^69^

In our studies, the nephroprotective effects of FMD were similar to those obtained with regular caloric restriction. Whether diet composition has an independent effect on AKI severity regardless of caloric amount is not clearly defined yet.

In conclusion, our study demonstrates that caloric restriction has a protective effect on the development of AKI and the subsequent progression to chronic kidney damage. This protective effect is, at least in part, mediated by a reduction in CCL2 levels, monocytic infiltration, and overall kidney inflammation. FMD also exerts a direct anti-proliferative and immunosuppressive effect on immune infiltrates and on monocytes especially. Altogether, these data support clinical studies testing the hypothesis that caloric restriction reduces the severity of AKI and transition from AKI to CKD.

### Limitations of the study

Although FMD demonstrated renoprotective effects in both acute and chronic settings, the mechanistic underpinnings remain incompletely resolved, as direct, immune-independent nephroprotective effects of fasting cannot be excluded. Additionally, all experiments were conducted in male mice, and potential sex-dependent responses to FMD were not addressed. The effects of repeated or long-term FMD cycles on renal structure, systemic metabolism, and immune function also remain unknown. Finally, translational applicability to humans requires further investigation, as dietary interventions may have species-specific effects and patient-specific challenges related to adherence, nutritional status, or comorbidities.

## Resource availability

### Lead contact

Further information and requests for resources and reagents should be directed to and will be fulfilled the lead contact, Paolo Cravedi (paolo.cravedi@mssm.edu).

### Material availability

This study did not generate new reagents.

### Data and code availability

• Data: Data reported in this paper are available from the lead contact upon request.
• Code: This paper does not report original code.
• Any additional information required to reanalyze the data reported in this work paper is available from the lead contact upon request.

## Acknowledgments

PC is supported by the 10.13039/100000002NIH grant R56AI132949.

## Author contributions

P. Molinari. and P.C. conceptualized the study. P. Molinari. and J.N. collected mouse samples. A.V., P. Molinari., K.F., and J.N. performed sample analyses. S.A. helped analyzing data. P. Malvezzi., A.V., P.C., V.L., G.C., C.A., and L.P. interpreted data. P.C. supervised the study. P.C. and P. Molinari. wrote the original draft of the manuscript. All authors read, edited, and approved the final manuscript.

## Declaration of interests

The authors declare no competing interests.

## STAR★Methods

### Key resources table

**Table:** 

REAGENT or RESOURCE	SOURCE	IDENTIFIER
**Antibodies**		

anti-CD45 BV605	Biolegend	103139; RRID: AB_2562341
anti-CD11b PerCP-Cy5. 5	Invitrogen	45-0112-82; RRID: AB_953558
anti-F480-PE	Biolegend	123110; RRID: AB_893486
anti-CD11c FITC	Invitrogen	11-0114-82; RRID: AB_464940
anti-LY6c APC	Invitrogen	17-5932-82; RRID: AB_1724153
anti-Ly6G Pe-Cy7	BioLegend	127618; RRID: AB_1877261
anti-Ki67 APC	Biolegend	652406; RRID: AB_2561930
anti-pAKT PeCy7	Cell Signaling	88106S; RRID: AB_2800113
PS6-Riboprotein AF647	Cell Signaling	4851S; RRID: AB_10695457
anti-IL1β PeCy7	Invitrogen	25-7114-82; RRID: AB_2573526
anti-TNFα PB	Biolegend	506318; RRID: AB_893639
anti-CCR2 APC	Biolegend	505910; RRID: AB_2566054
Fab Fragment Goat Anti-Mouse IgG	Jackson ImmunoResearch	AB_2338476
Goat anti-mouse KIM-1 IgG	Biotechne	AF1817; RRID: AB_2116446
Rabbit anti-mouse VCAM1 IgG	Invitrogen	MA5-31965; RRID: AB_2809259
Rabbit anti-alpha smooth muscle IgG	abcam	ab5694; RRID: AB_2223021
Rat anti Mouse F4/80 IgG	Invitrogen	14-4801-82; RRID: AB_467558
Lotus Tetragonolobus Lectin	Vector Laboratories	FL-1321-2; RRID: AB_2336559
Anti-Rabbit IgG antibody	Invitrogen	A31573; RRID: AB_2536183
Donkey Anti-Goat IgG	Invitrogen	A11056; RRID: AB_2534103
DAPI	Invitrogen	P36931

**Chemicals, peptides, and recombinant proteins**		

Folic acid	Sigma-Aldrich	F7876
Aristocholic acid	Sigma-Aldrich	A5512
*Ad libitum*/Low caloric diet	Inotiv	7912
Fasting-mimicking diet	XXX	XXX
CCR2 chemokine receptor antagonist	Tocris Bioscience/Bio-Techne	2517
*TNFα*	ThermoFisher Scientific	Mm00443258_m1
*IL1β*	ThermoFisher Scientific	Mm00434228_m1
*Tgfβ1*	ThermoFisher Scientific	Mm01178820_m1
*IL-6*	ThermoFisher Scientific	Mm00446190_m1
*Ccl2*	ThermoFisher Scientific	Mm00441242_m1
*IL33*	ThermoFisher Scientific	Mm00505403_m1
*Gapdh*	ThermoFisher Scientific	Mm99999915_g1
Paraformaldehyde 16%	Electron Microscopy Sciences	15710-S
Tissue-Tek O.C.T.	Sakura	4583
Trizol	Invitrogen	498806

**Critical commercial assays**		

Urea Nitrogen (BUN) Colorimetric Detection Kit	ThermoFisher Scientific	CAT: EIABUN
Multi Tissue Dissociation Kits	Miltenyi Biotec	130-110-204
Mouse CCL2/JE/MCP-1 DuoSet ELISA	Bio-Techne	DY479

**Deposited data**		

Raw and analyzed data	This paper	Shared upon request by the lead contact
Histological staining techniques data	This paper	Shared upon request by the lead contact

**Experimental models: Organisms/strains**		

C57BL/6J (B6) wild type	Jackson Laboratory	Strain: 000664
BALB/c wild type	Jackson Laboratory	Strain: 000651

**Software and algorithms**		

GraphPad 10.1.2	Prism	https://www.graphpad.com/
FlowJo 10	BD	https://www.flowjo.com/
ImageJ	National Institutes of Health	https://imagej.nih.gov/ij/

### Experimental model and study participant details

#### Animals

BALB/c and C57BL/6J (B6) wild type (WT) male mice were purchased from the Jackson Laboratory (Bar Harbor, ME). Only male mice were used due to the known inter-gender variability in response to renal injury and in consideration of similar previous models of renal disease.^45^^,^^46^^,^^69^^,^^70^ Animal study protocol was approved by the institutional animal care and use committee at Icahn School of Medicine at Mount Sinai (New York, NY; IACUC ID PROTO202000116). The reference number of the Office of Laboratory Animal Welfare approved Animal Welfare Assurance of Icahn School of Medicine at Mount Sinai is D16-00069 (A3111-01).

#### *Ad libitum*, FMD and low caloric diets

At the age of 8–12 weeks, mice were randomly allocated to *ad lib* or FMD starting one week before AA injection, until the day of sacrifice (14^th^ day or 35^th^ day). Investigators in charge of the histological and immunological analyses were blinded to the treatment arm.

*Ad libitum* (*ad lib*) chow consisted of irradiated TD.7912 rodent chow, containing 15.69 kJ/g of digestible energy (animal-based protein 3.92 kJ/g, carbohydrate 9.1 kJ/g, fat 2.67 kJ/g).

The FMD diet consisted of two different components designated as day 1 diet and day 2–5 diet that will be administered in this order. Based on our preliminary data, mice are expected to consume all the supplied food on each day of the FMD regimen with no signs of food aversion. Briefly the diet is made by a mix of various low-calorie broth powders, a vegetable medley powder, extra virgin olive oil, and essential fatty acids mixed with hydrogel as described elsewhere.^71^

On day 1, mice consume about 50% of their normal caloric intake (3.3g of FMD, 7.87 kJ/g). On days 2–5 mice consume about 10% of their normal caloric intake (2g of FMD, 1.51 kJ/g). On average, mice consume 11.07 kJ (plant-based protein 0.75 kJ, carbohydrate 5.32 kJ, fat 5 kJ) on each day of the FMD regimen. After the end of FMD, we will supply irradiated TD.7912 rodent chow (Harlan Teklad) *ad lib* for 2 days before starting another FMD cycle. A cycle was therefore composed by 5 days of FMD and 2 *ad lib* diet.

Prior to supplying the FMD, animals will be transferred into fresh cages to avoid feeding on residual chow and coprophagy. More details on the FMD composition are available in.^72^ Body weight was measured 3 times per week.

The low caloric group consisted in decreasing to quantity of normal chow to reach the same caloric intake compared to FMD, i.e., 3.3g (7.87 kJ/g) on day 1 and 0.6 g/day (1.51 kJ/day) on days 2–5.

### Method details

#### Aristolochic acid injection

BALB/c male mice (8–12 weeks of age) were given a single intraperitoneal injection of AA at the dose of 5 mg/kg (Sigma-Aldrich #102501333) dissolved in saline, and they were euthanized 14 or 35 days later, while B6 mice, more resistant to AA-induced nephropathy, were given 3 IP injections every other day (5mg/kg each) and euthanized 14 days after.^45^^,^^69^

#### Folic acid injection

BALB/c male mice (8–12 weeks of age) were given a single intraperitoneal injection of Folic acid (FA) at the dose of 250 mg/kg (Sigma-Aldrich #102515729) dissolved in Sodium Bicarbonate (100mM), and they were euthanized 7 days later.^46^

#### BUN and serum creatinine measurement

Blood samples for serum were collected before AA injection and weekly until the day of the sacrifice. Serum was isolated from whole blood by centrifuging samples in BD Microtainer (BD Biosciences, #365967) at 12.000 g × 10 min. Serum samples were analyzed immediately or stored at −20C^o^. Multiple freeze thaw cycles were avoided. Serum Blood Urea Nitrogen (BUN) was quantified in serial blood collections using colorimetric detection kit from Thermo Scientific according to the manufacturers’ instructions (Urea Nitrogen (BUN) Colorimetric Detection Kit, Product# EIABUN, Thermo Fisher Scientific). Serum creatinine was quantified by liquid chromatography with tandem mass spectrometry (LC-MS/MS).^73^

#### Renal histology

Mice were anesthetized with a 100-*μ*L I.P. injection of a solution made of sterile ketamine (16 mg/mL) and xylazine (7 mg/mL) in PBS (Corning #46-13-CM), and then, received intracardiac perfusion of PFA (Electron Microscopy Sciences #15710-S) fixate at 4% in PBS at a rate of 8–10 mL/min. Kidneys were harvested and embedded in paraffin or frozen in optimal cutting temperature (OCT) compound (Tissue-Tek O.C.T.; Sakura #4583).

##### Light microscopy

Paraffin-embedded kidney sections (3 *μ*m) were stained with H&E (hematoxylin and eosin stained). Sections were scored for ATN and interstitial inflammation. Score for ATN and inflammation ranged from 0 to 5, depending on the percentage of tissue involved (0: absent - 1: <20% - 2: ≥20%, <40% - 3: ≥40%, <60% - 4: ≥60%, <80% - 5: ≥80%). Scoring was performed by two independent investigators. In case of disagreement between investigators, we used the average score. We derived our scoring system of tubulointerstitial damage based on scores previously used in similar disease models.^74^^,^^75^

##### Immunofluorescence

O.C.T.-preserved cryosections (5 μm thick) were fixed with PFA 4% (Electron Microscopy Sciences #15710-S) for 15 min and then washed for 5 min × 3 times in 1X PBS (Corning #46-0.13-CM). Samples were incubated with AffiniPure Fab Fragment Goat Anti-Mouse IgG (H + L) (Jackson Immuno #115-007-003). Then samples were incubated with primary antibodies, goat anti-mouse KIM-1 IgG polyclonal antibody, 1:200 dilution in 0.1% BSA (Biotechne, #AF1817), Rabbit anti-mouse VCAM1 IgG monoclonal antibody (Invitrogen #MA5-31965), 1:100 dilution in 0.1% BSA, Rabbit anti-alpha smooth muscle polyclonal antibody (abcam, #ab5694), 1:500 dilution in 0.1% BSA and Rat anti Mouse F4/80 polyclonal antibody (Invitrogen #14-4801-82), 1:100 dilution in 0.1% BSA for 60 min at room temperature. Thereafter, tissues were washed again with 1X PBS 5 min × 3 times. Tissue were then stained with secondary antibodies., LTL (Lotus Tetragonolobus Lectin, Vector Laboratories # FL-1321-2) was used to mark PTEC, 1:200 dilution in 0.1% BSA; Donkey Anti-Goat IgG, AF546 polyclonal antibody was used to bind Anti-KIM-1, 1:200 dilution in 0.1% BSA, (Invitrogen #A11056); Anti-Rabbit IgG AlexaFluor647 was used to mark Anti-Ki67 antibody, 1:200 dilution in 0.1% BSA (Invitrogen #A31573). Sections were then washed with 1X PBS 5 min × 3 times. After that, Nuclei were counterstained with 4′,6-diamidino-2-phenylindole (DAPI) mounting media (ProLong™ Gold Antifade Mountant with DNA Stain, Invitrogen #P36931).

Images were acquired on a Zeiss widefield Axio Imager.Z2(M). Mean fluorescence intensity of various target antigens was quantified by using ImageJ software (National Institutes of Health, Bethesda, MD) within contour masks created on the LTL-stained image to identify PTECs. MFI was normalized as follows: (MFI-Background or Isotype MFI).

#### Flow cytometry analysis

At sacrifice, spleens and kidneys were harvested and individually analyzed. Splenocytes were manually isolated. Kidneys cells were isolated by processing them in a solution made with RPMI medium (Gibco, #11875-093) and Multi tissue dissociation kit enzymes (Mylteni Biotec, #130-110-101). The kidney-enzyme solution was then transferred to GentleMacs C-tubes (Mylteni Biotec #130-093-237) and processed for 1 h in GentleMacs OCTO dissociator with heaters (Mylteni Biotec). Both spleens and kidneys were treated with ACK lysing buffer (Quality Biologicals #118-156-101) and subsequently filtered to discard residual red blood cells. Single cell suspensions (1 to 3×10^6^) were first incubated with FCblock (BD Biosciences #553142) 30 min 4 C° and then stained with fluorescent-labelled monoclonal antibodies for cell surface antigens and incubated for 30 min at 4°C. Monocytes and macrophages were stained using anti-CD45 BV605 (Biolegend #103139), anti-CD11b PerCP-Cy5. 5 (Invitrogen #45-0112-86), anti-F480-PE (Biolegend #123110), anti-CD11c FITC (Invitrogen #11-0114-82), anti-LY6c APC (Invitrogen #17-5932-82), anti-Ly6G Pe-Cy7 (BioLegend #127618). To evaluate Ki67 expression, cells were permeabilized with 2mL of 70% ice-cold methanol (−20C°) and incubated for 1h at −20C°. Ki67 was stained using anti-Ki67 APC (Biolegend #652406) and incubated for 30 min RT in the dark. To evaluate mTor pathway proteins cells were fixed with PFA 4% methanol-free (Cell Signaling Technology #47746) for 15 min RT. Afterward cells were permeabilized with ice-cold methanol (−20C°) for 30 min on ice. Cells were then stained using anti-pAKT PeCy7 (Cell Signaling #88106S) and anti PS6-Riboprotein AF647 (Cell Signaling #4851S) for 1h RT in the dark. Monocytes interleukin production was evaluated by plating splenocyte cells in a 96-well round bottom culture plate. Cells were stimulated with LPS (Sigma Aldrich #L2880) and incubated for 1h at 37°C and 5% CO_2._ After, cells were incubated for 4h with GolgiPlug (BD Biosciences #555029) at 37°C and 5% CO_2._ Cells were fixed and permeabilized using eBioscience FOXP3/Transcription Factor Staining Buffer Set (Thermo Fisher Scientific #00-5523-00), for 30 min RT. Fixed and permeabilized cells were stained with anti-IL1β PeCy7 (Invitrogen #25-7714-82), anti-TNFα PB (Biolegend #506318) and anti-CCR2 APC (Biolegend #505910) for 30 min RT in the dark. At least 50,000 cells were acquired per sample. Samples were acquired on a BD LSRII, on a three-laser Canto II (BD Biosciences) flow cytometer and analyzed with FlowJo (https://www.flowjo.com) software (Ashland, OR).

#### Real-time quantitative RT-PCR

Samples for RNA extraction were preserved using Trizol (Invitrogen #498806), stored in −80 and processed with a tissue omogeneizer after thawing. After homogenization RNA was isolated using a gradient of alcohols (1,3 bromo-mercapto-ethanol – isopropanol −70% ethanol). RNA quality was determined by analysis of the A260/A280 ratio and concentration was detected by Nanodrop (ThermoFisher Scientifics NanoDrop 2000). cDNA was synthesized using reverse transcription reagent (Applied Biosystems #4368814). Real-time PCR assays using the TaqMan universal PCR Master Mix and primer sets for mouse *TNFα* (Mm00443258_m1), *IL1β* (Mm00434228_m1), *Tgfβ1* (Mm01178820_m1), *IL-6* (Mm00446190_m1), *Ccl2* (Mm00441242_m1), *IL33* (Mm00505403_m1), and *Gapdh* (Mm99999915_g1) genes were purchased from ThermoFisher. PCR was performed on an Applied Biosystems 7500 Fast system. All experiments were performed at least in duplicate, and *TNFa*, *IL-1β*, *Tgf1β*, *IL-6*, and *CCL2* gene expression were normalized to housekeeping gene *Gapdh* and WT mice with no disease or specific diet.

#### Serum CCL2 measurement

Measurement of the chemokines C-C motif chemokine ligand 2 (CCL2) in the serial blood collections was achieved using the CCL2/JE/MCP1 (R&D Systems #DY479) DuoSet ELISA kits. Serum was isolated from whole blood by centrifuging samples in BD Microtainer (BD Biosciences, #365967) at 12,000 g × 10 min. Serum samples were analyzed immediately or stored at −20°C. Multiple freeze-thaw cycles were avoided. The assays were performed according to the manufacturer’s instructions in a 96-well ELISA plate. A microplate reader (uQuant, Biotech) was used to measure absorbance at 450 nm.

#### CCR2 inhibitor

Highly selective CCR2 chemokine receptor antagonist (6-Methyl-1′-[2-(5-methyl-2-phenyl-4-oxazolyl)ethyl]-spiro[4H-3,1-benzoxazine-4,4′-piperidin]-2(1H)-one) was purchased from Tocris Bioscience (Tocris Bioscience/Bio-Techne #2517). The drug was diluted in DMSO (Fischer Scientific #67-68-5) up to 10% as per manufacturer instructions and incubated for 12-24h at 40-45°C on a horizontal shaker at 300rpm to achieve complete dissolution. The drug was injected IP at a dosage of 2mg/Kg, every day from the day of AA injection till the day of the sacrifice (14 days), as per previous reports.^76^

### Quantification and statistical analysis

Continuous variables were reported as mean ± SE. Comparisons between groups distributions were carried out with unpaired T-test (normal distributions), Mann-Whitney (non-normal distributions) test, repeated measures ANOVA model was used for multiple comparisons in time (normal distributions) or Kruskal-Wallis test (non-normal distributions) and one-way ANOVA was used for multiple group comparisons. Analyses comparing fold changes vs. control were carried out using one sample T-test. Pearson correlations and simple linear regression analyses were used to correlate continuous variables. *p* values < 0.05 were considered significant. All statistical analyses were performed using Prism, version 9, for Windows (GraphPad Software Inc.).
