# Supplementary material for: Caloric restriction protects from acute and chronic kidney injury by inhibiting monocyte recruitment
Source: iScience. 2025 Jul 11;28(8):113094. doi: 10.1016/j.isci.2025.113094 (PMC12314339; doi:10.1016/j.isci.2025.113094)
Supplement: Document S1. Figures S1–S9 [file mmc1.pdf]

iScience, Volume *28*

Supplemental information

Caloric restriction protects from acute and chronic kidney injury by inhibiting monocyte recruitment

Paolo Molinari, Alberto Verlato, Johan Noble, Sara Alibrandi, Sofia Bin, Katja Ferrari, Paolo Malvezzi, Carlo Alfieri, Giuseppe Castellano, Laura Perin, Valter Longo, and Paolo Cravedi

Content Summary

- Figure S1. Study design.
- Figure S2. Weight changes in controls, FMD, Low Cal and *ad lib* mice.
- Figure S3. Renal function and histological damages post AA in FMD and Low Cal diet.
- Figure S4. Characterization of cytokine production from kidney infiltrating monocytes in mice.
- Figure S5. Characterization of kidney and spleen monocytic inflammatory infiltrate in ad lib and FMD mice.
- Figure S6. Renal macrophage infiltration in AA-injected mice on FMD, *ad lib* diet, or after CCR2i treatment.
- Figure S7. mTOR, PS6-Riboprotein and Ki67 expression in kidney and spleen immune and parenchymal cells
- Figure S8. Fibrosis and macrophages infiltration in AA-induced chronic kidney disease.
- Figure S9. Characterization of kidney monocytic infiltrate and pro-inflammatory cytokines in ad lib and FMD mice after delayed FMD start

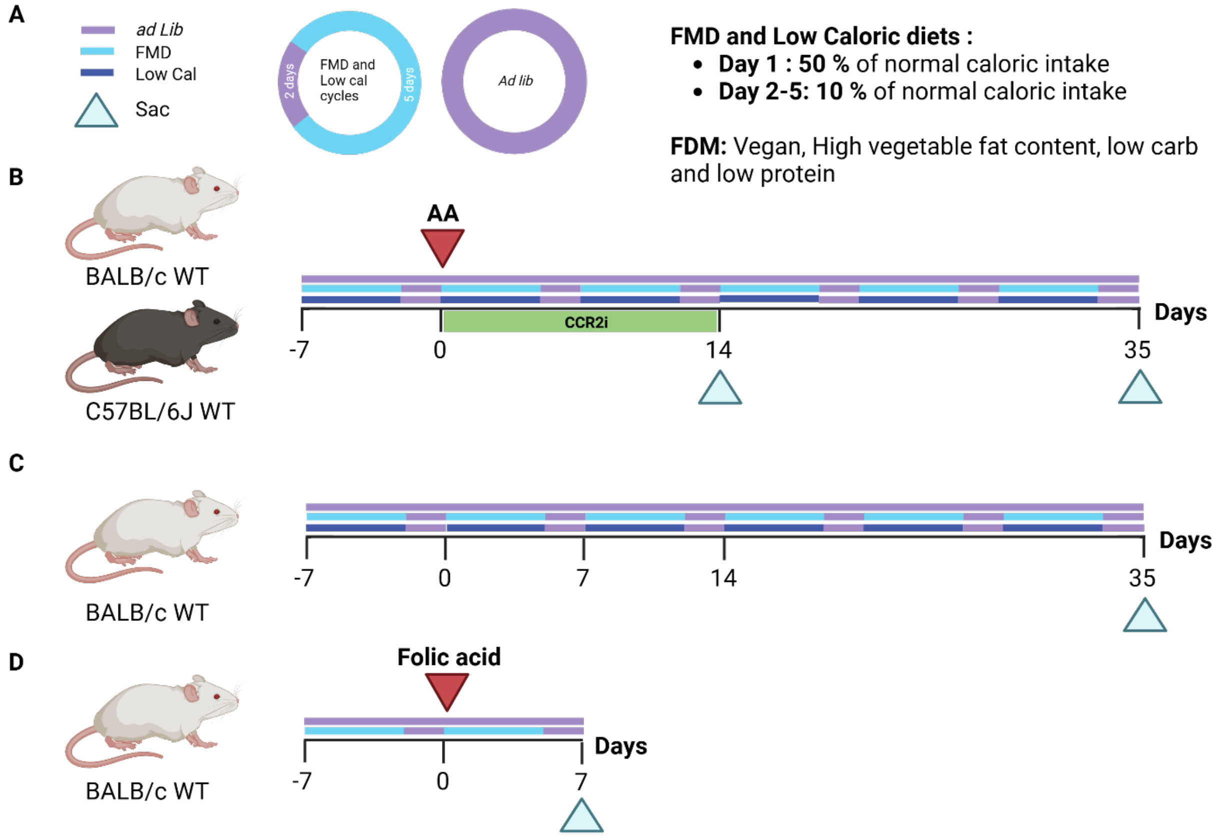

Figure S1. Study design. (**A**) Fasting mimicking diet (FMD) or caloric restriction (Low Cal) cycle and *ad lib* diets. (**B**) WT male BALB/c or C57BL/6J mice received AA and were sacrificed at 14 days (peak of AKI) or at 35 days thereafter. In this model, CCR2 inhibitor (CCR2i) or vehicle was administered every day from day 0 after AA injection to day 14 + FMD or *ad lib* diet (diet allocation started on day -7 before AA injection). (**C**) WT male BALB/c mice received AA and were sacrificed at 35 days thereafter. Diet allocation started on day -7 before AA injection or at day 14 after AA injection. (**D**) WT male BALB/c mice received folic acid and were sacrificed at 7 days thereafter. FMD or *ad lib* diet was started at day -7 before folic acid injection.

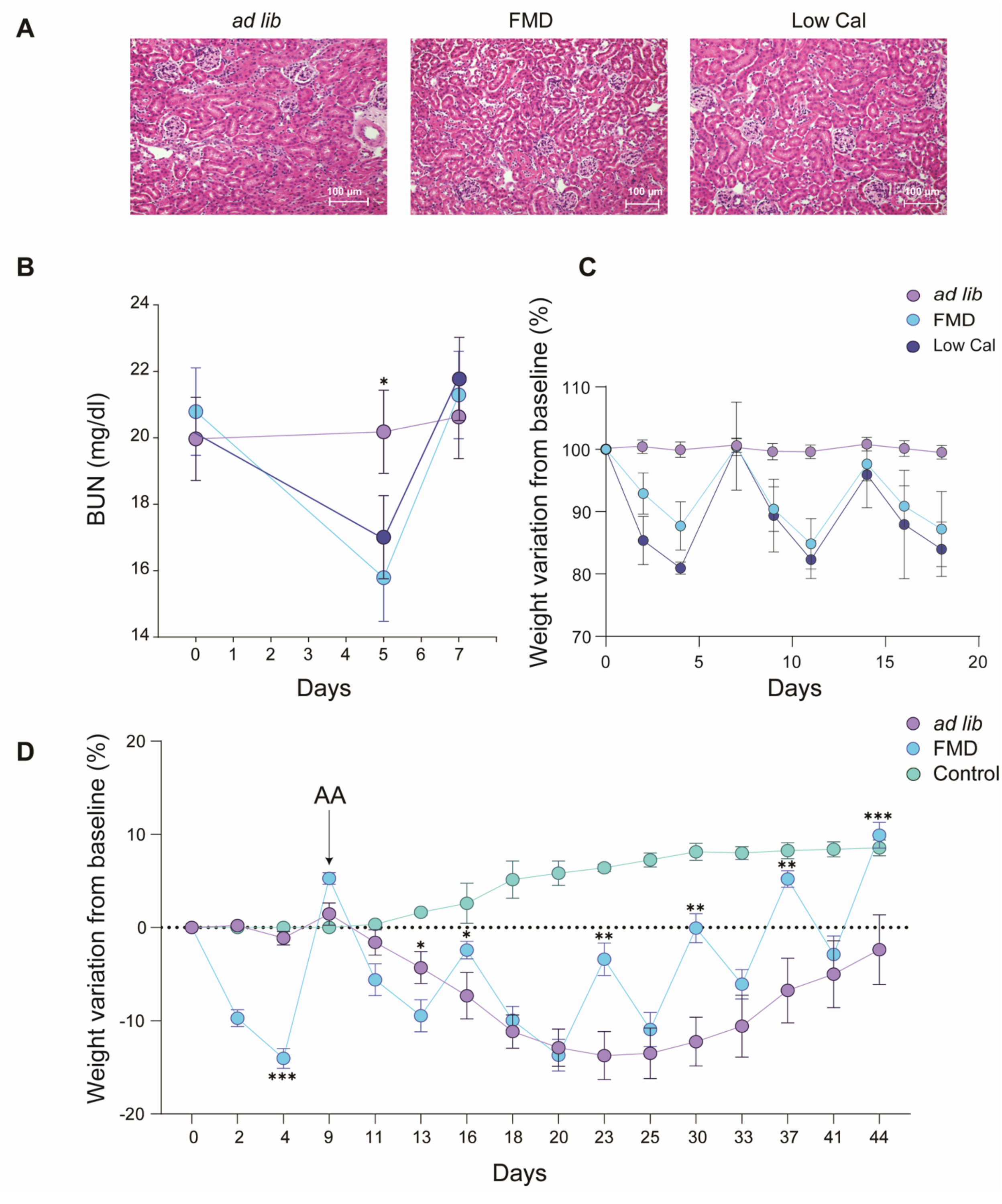

Figure S2. Weight changes in controls, FMD, Low Cal and *ad lib* mice. (**A**) Representative brightfield images (H&E) of kidney sections from B6 mice on ad lib, FMD, or Low Cal diet for 14 days. At Day 0, mice were injected with the vehicle solution used for AA (**B**) Blood urea nitrogen at serial time points after ad lib or one FMD cycle (*ad lib* n=5, FMD n=5, Low Cal n=6). (**C**) Weight changes after 3 cycles in *ad lib*, FMD and Low Cal diets mice (*ad lib* n=5, FMD n=5, Low Cal n=6). (**D**) Weight changes in controls (*ad lib*, not subjected to AA injection), and in *ad lib* and FMD mice at different time points after AA injection (*ad lib* n=13, FMD n=18). Two-way ANOVA was used to assess statistical

significance at different time points. * p<0.05, ** p<0.01, *** p<0.001, **** p<0.0001 vs. *ad lib*; ns: not significant.

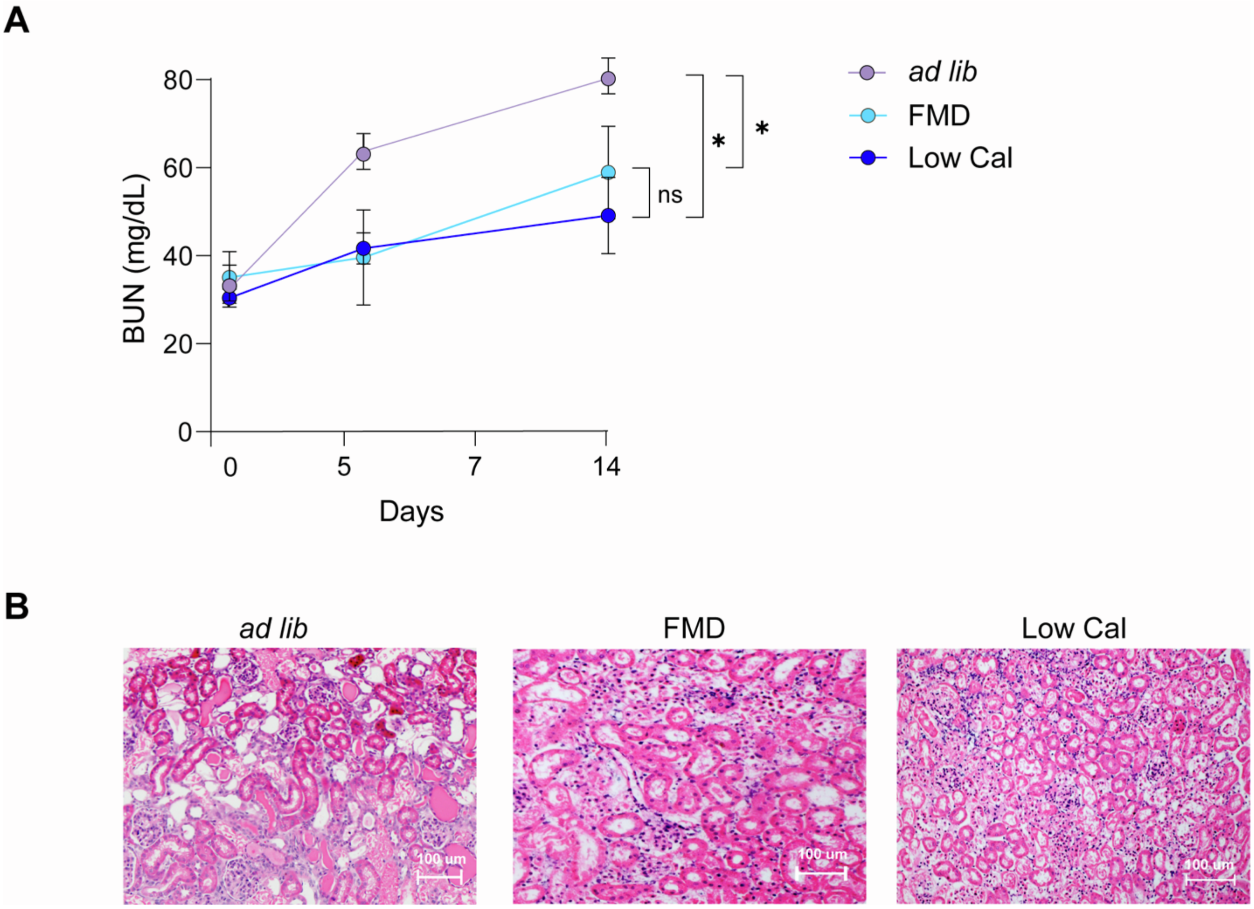

Figure S3. Renal function and histological damages post AA in FMD and Low Cal diet. **A**) Blood urea nitrogen at serial time points after 1 FMD cycle and Low Cal diet pre-AA injections and 2 cycles of FMD cycle and Low Cal diet post-AA injections (*ad lib* n=5, FMD n=4, Low Cal n=4). **B)** Representative brightfield images (H&E) of ad lib, FMD and Low Cal diet of kidney cortical tubular sections at day 14 after AA injection. Two -way ANOVA was used to assess statistical significance at different time points. ns: not significant.

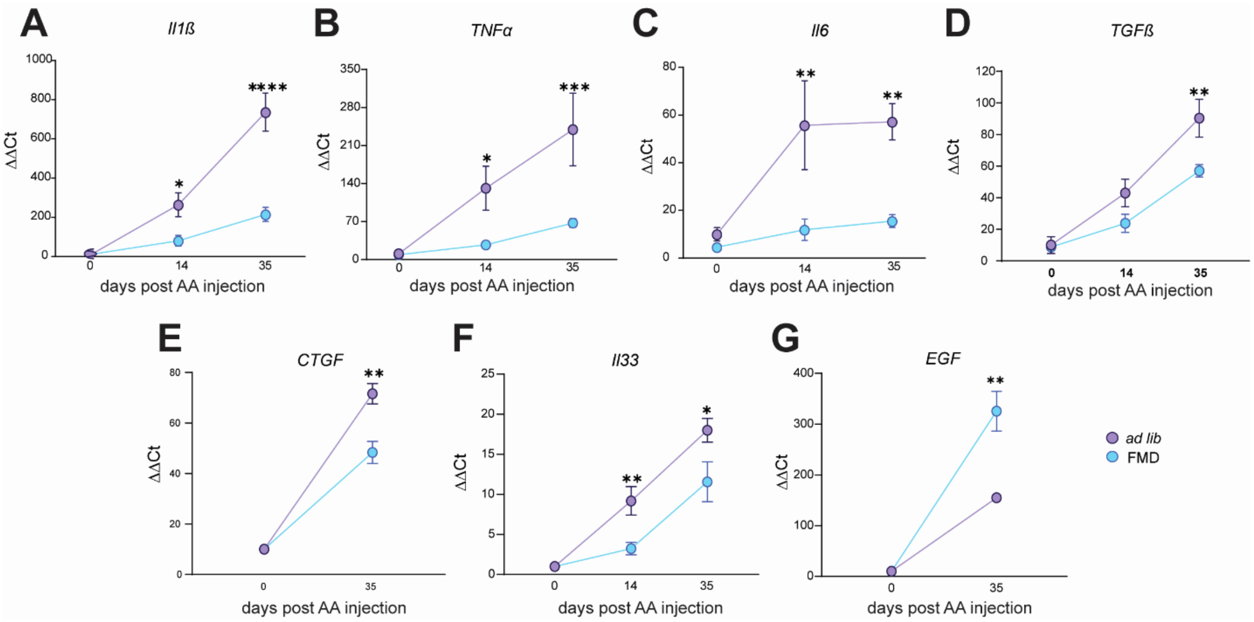

Figure S4. Characterization of cytokine production from kidney infiltrating monocytes in mice. (**A-D**) *Il1β, TNFα, IL6*, and *TGFβ* gene expression in the kidneys at 0, 14, and 35 days after AA injection in *ad lib* and FMD mice and (**D-G**) *CTGF, IL-33* and *EGF* at 0, 14 days and 35 days after AA injection in *ad lib* and FMD mice (*ad lib*, n= 7 at 14 days and n=6 at 35 days and for FMD, n=9 at 14 days and n=8 at 35 days). FMD was started 7 days before AA injection. Repeated measures ANOVA model was used to assess statistical significance at different time points. * $p<0.05$, ** $p<0.01$, *** $p<0.001$, ****$p<0.0001$ vs. *ad lib* at the same time point; ns: not significant.

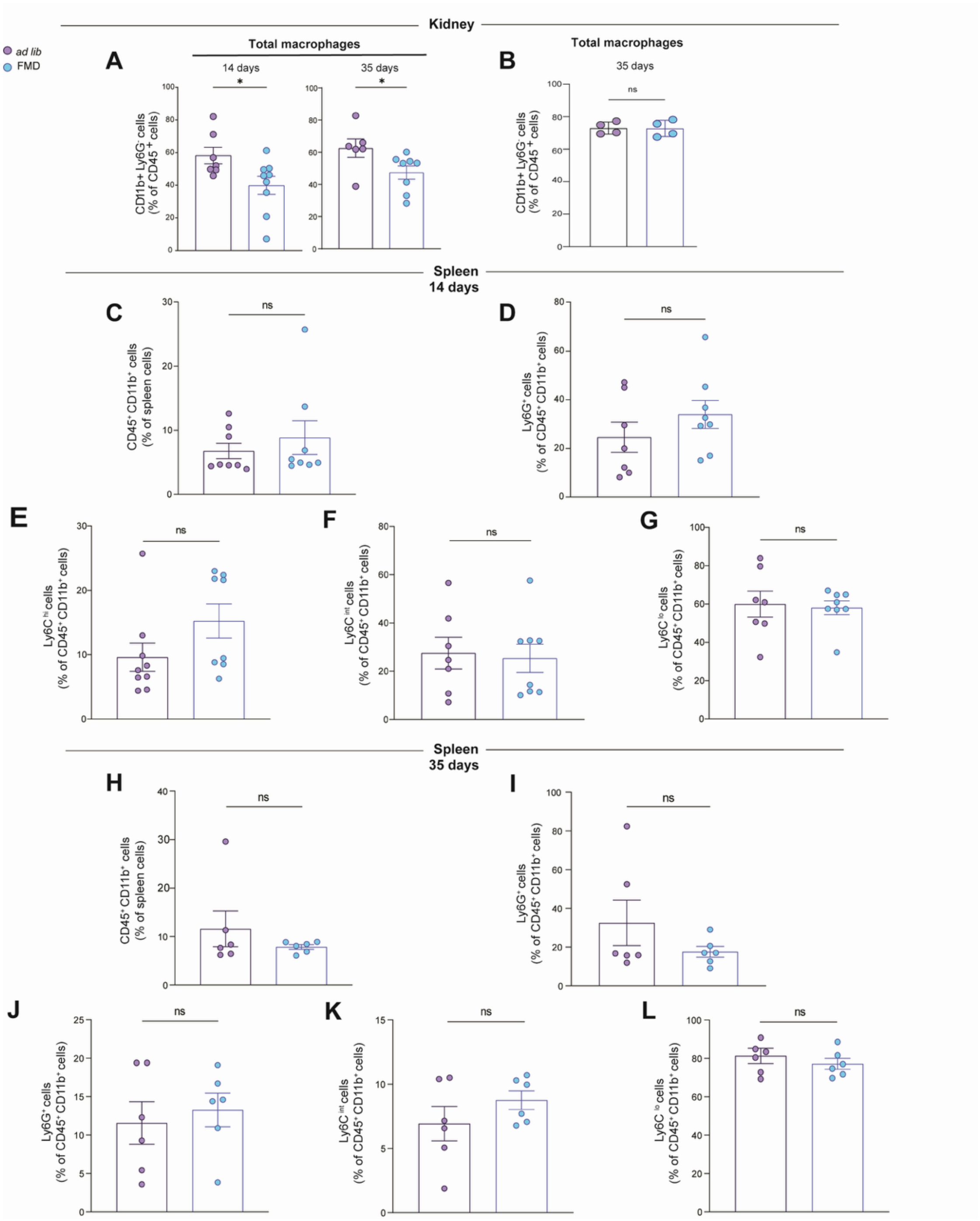

Figure S5. Characterization of kidney and spleen immune cells in *ad lib* and FMD mice. (A) kidney quantification (bar plot) of CD45$^+$CD11b$^+$ Ly6G$^-$macrophages at 14 and 35 days after AA in *ad lib* and FMD mice and of **(B)** CD45$^+$CD11b$^+$Ly6g$^-$ macrophages at 35 days in *ad lib* and FMD mice without AA**.** Quantification of splenic (**C**) CD45$^+$ CD11b$^+$ cells, (**D**) CD11b$^+$ Ly6g$^+$ cells, (**E**) CD11b$^+$ Ly6c^{hi} cells, (**F)** CD11b$^+$ Ly6c^{int} cells, and (**G)** CD11b$^+$ Ly6c^{lo} cells at 14 days after AA injection in *ad lib* and FMD mice. Quantification of splenic (**H**) CD45$^+$ CD11b$^+$ cells, (**I**) CD11b$^+$ Ly6g$^+$ cells, (**J**) CD11b$^+$ Ly6c^{hi} cells, (**K**) CD11b$^+$ Ly6c^{int} cells, and (**L**) CD11b$^+$ Ly6c^{lo} cells at 14 days after AA injection in *ad lib* and FMD mice. *Ad lib*, n = 7 at 14 days and n= 6 at 35 days, FMD, n = 9 at 14 days and n = 8 at 35 days. T-test was used to compare distributions between groups at the same time point * p<0.05 vs. *ad lib*; ns: not significant.

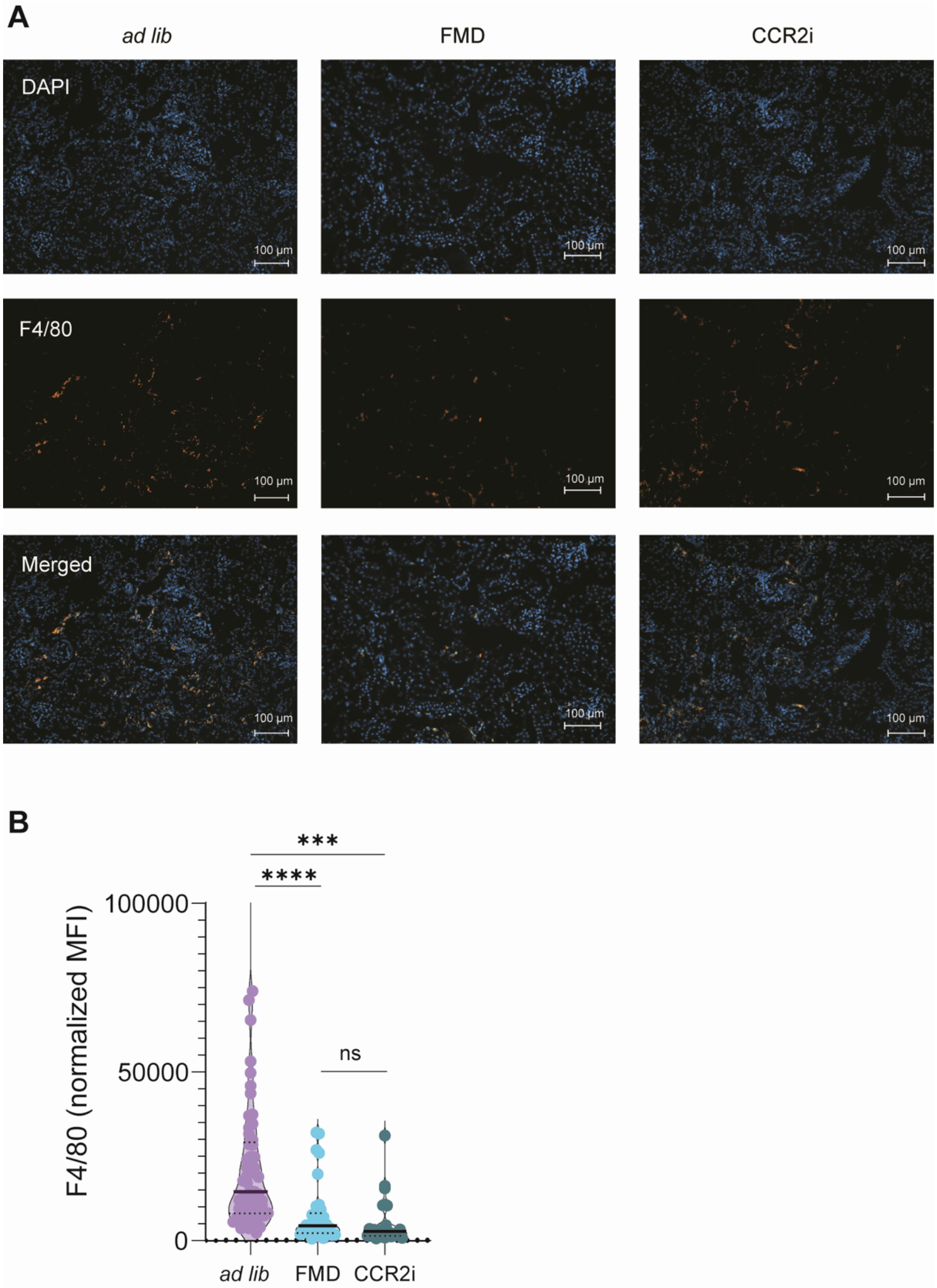

Figure S6. Renal macrophage infiltration in AA-injected mice on FMD, *ad lib* diet, or after CCR2i treatment. (A) Representative IF images of kidney sections from B6 mice on *ad lib* or FMD, or CCR2i treatment, at 15 days after AA injection. DAPI is represented in blue and F4/80 in orange. **(B)** F4/80 normalized MFI quantification around the cortical tubules at 15 days (ad lib, n=2, FMD, n=2 and CCR2i, n=2). One-way ANOVA was used to compare distributions between the 3 groups. * $p<0.05$, ** $p<0.01$, *** $p<0.001$, **** $p<0.0001$; ns: not significant.

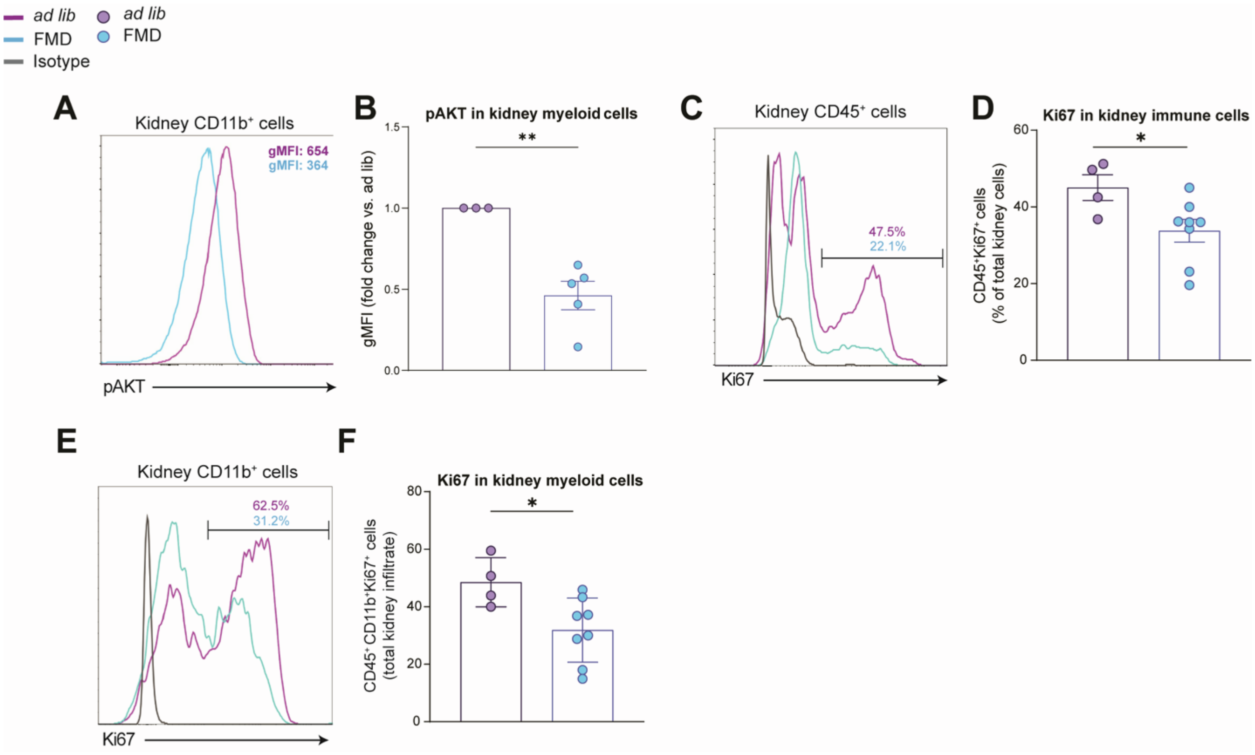

Figure S7. mTOR, PS6-Riboprotein and Ki67 expression in kidney and spleen immune and parenchymal cells (**A**) Representative histogram of pAKT and (**B**) gMFI quantification in kidney CD11b+ cells from *ad lib* (n=3) vs FMD mice (n=5). (**C**) Representative MFI histogram and (**D**) quantification of $Ki67^+$ positivity in kidney $CD45^+$ immune cells from *ad lib* (n=4) vs FMD mice (n=8). (**E**) Representative MFI histogram and (**F**) quantification of $Ki67^+$ positivity in kidney $CD11b^+$ cells from *ad lib* (n=4) vs FMD mice (n=8). T-test was used to compare distributions between groups at the same time point. * p<0.05, ** p<0.01, *** p<0.001, ****p<0.0001 vs. *ad lib* at the same time point; ns: not significant. Data were reported as mean±SEM.

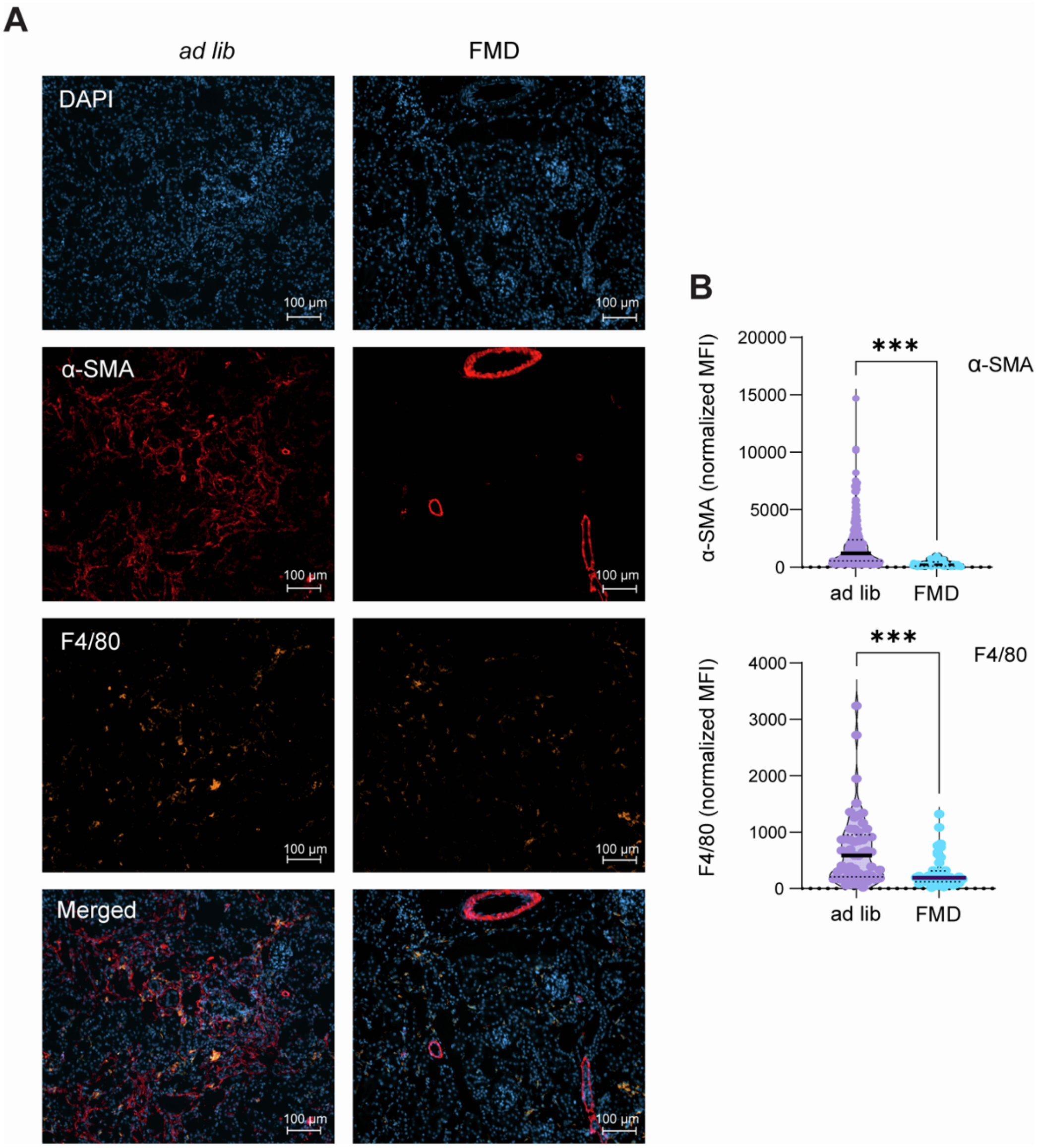

Figure S8. Fibrosis and macrophages infiltration in AA-induced chronic kidney disease. (A) Representative IF images of DAPI (Blue), α-SMA (Red) and F4/80 (Orange), in B6 mice on *ad lib* or FMD at 35 days after AA injection. **(B)** α-SMA and F4/80 normalized MFI quantification in the interstitium and around cortical tubules at 35 days (ad lib, n=2, FMD, n=2). T-test was used to compare distributions between groups at the same time point. * $p<0.05$, ** $p<0.01$, *** $p<0.001$, **** $p<0.0001$; ns: not significant.

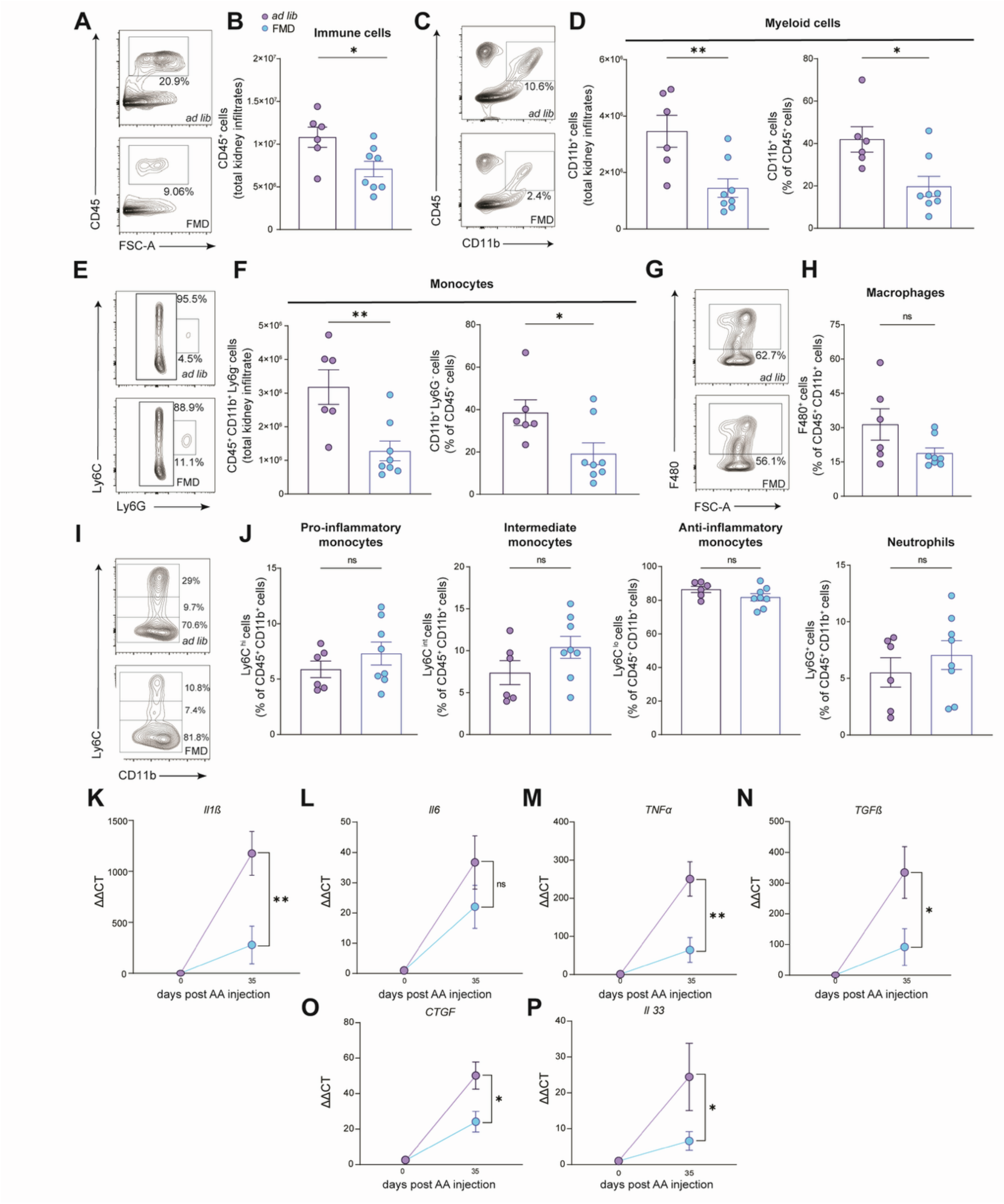

Figure S9. Characterization of kidney monocytic infiltrate and pro-inflammatory cytokines in ad lib and FMD mice after delayed FMD start. Mice started FMD or continued *ad lib* diet at 14 days after AA injection (peak of AKI). (**A**) Representative contour plot at day 14 and (**B**) quantification of kidney $CD45^+$,(**C, D**) of $CD45^+CD11b^+$ cells, (**E, F**) of $CD45^+CD11b^+Ly6g^-$ cells, (**G,H**) of $F480^+CD11b^+$ cells, and (**I, J**) of $CD11b^+Ly6C$ high (^{hi}), intermediate (^{int}), low and $CD11b^+Ly6G^+$ cells at 35 days after AA injection (ad lib n=6, FMD n=8). (**K-P**) *Il1β, Il6, TNFα*, *TGFβ, CTGF and IL33* gene expression in the kidneys at 0 and 35 days after AA injection (ad lib n=6, FMD n=8). Repeated measures ANOVA model was used to assess statistical significance at different time points (Panel K - P). T-test was used to compare distributions between groups at the same time point.* p<0.05, ** p<0.01, vs. ad lib at the same time point; ns: not significant.
